# Deciphering Age‐Dependent ECM Remodelling in Liver: Proteomic Profiling and Its Implications for Aging and Therapeutic Targets

**DOI:** 10.1111/cpr.70087

**Published:** 2025-06-19

**Authors:** Juan Liu, Qingru Song, Chen Li, Jiexin Yan, Ni An, Wenzhen Yin, Jinmei Diao, Yuxin Su, Yunfang Wang

**Affiliations:** ^1^ Hepato‐Pancreato‐Biliary Center, Beijing Tsinghua Changgung Hospital, School of Clinical Medicine, Tsinghua Medicine Tsinghua University Beijing China; ^2^ Key Laboratory of Digital Intelligence Hepatology, Ministry of Education, School of Clinical Medicine, Tsinghua Medicine Tsinghua University Beijing China; ^3^ Beijing Advanced Center of RNA Biology (BEACON) Peking University Beijing China; ^4^ College of Chemistry and Life Sciences Beijing University of Technology Beijing China; ^5^ The Third Affiliated Hospital of Naval Medical University Shanghai China; ^6^ Clinical Translational Science Center, Beijing Tsinghua Changgung Hospital Tsinghua University Beijing China

**Keywords:** extracellular matrix (ECM), liver aging, Lumican, Matrisome, proteomics

## Abstract

Aging is characterised by progressive structural and functional changes in the liver, with the extracellular matrix (ECM) playing a key role in modulating these changes. Our study presents a comprehensive proteomic analysis of the liver ECM across different age stages, uncovering significant age‐related changes. Through the identification of 158 ECM proteins in decellularised rat liver scaffolds, we reveal the intricate relationship between ECM composition and liver maturation, as well as the decrease in regenerative capacity. Lumican was identified as a critical regulator with heightened expression in neonatal livers, which is associated with enhanced hepatocyte proliferation and maintenance of stem cell characteristics. Temporal expression analysis distinguished four distinct clusters of ECM proteins, each reflecting the liver's functional evolution from early development to old age. Early developmental stages were marked by proteins essential for liver growth, while adulthood was characterised by a robust ECM supporting metabolic functions. Middle age showed a regulatory shift towards protease balance, and later life was associated with haemostasis‐related processes. Our findings underscore the multifaceted role of the ECM in liver health and aging, offering potential opportunities for therapeutic intervention to counteract age‐induced liver dysfunction. This study provides a foundational understanding of ECM dynamics in liver aging and sets the stage for the development of innovative strategies to mitigate the effects of age‐related liver decline.

## Graphical abstract

This study reveals age‐dependent remodelling of the liver extracellular matrix (ECM), identifying Lumican as a key regulator of hepatocyte proliferation and stemness in foetal development. Dynamic ECM shifts influence liver function, offering insights into therapeutic strategies for aging‐related hepatic decline.

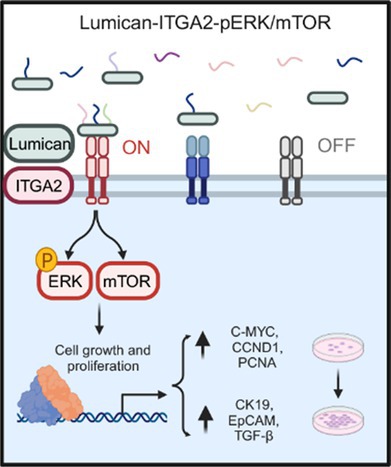

## 1 Introduction

The phenomenon of organismal aging is an inescapable aspect of the human life process that has garnered significant attention [1, 2, 3, 4]. The liver, serving as the body's largest metabolic organ, not only shoulders a myriad of pivotal roles encompassing metabolism, detoxification and storage but also assumes a paramount role in the maintenance of bodily homeostasis and immune regulation [5]. Hence, it is regarded as a pivotal linchpin for the preservation of overall physiological well‐being. With the progression of age, the liver exhibits degenerative alterations, characterised by exacerbated inflammation and fibrosis, a waning regenerative capacity and a compromised ability to ameliorate damage [6]. These cumulative changes culminate in an elevated predisposition to the onset of associated diseases [7, 8, 9].

Throughout the annals of research, the primary focus in the study of liver aging has centred on delineating the intricacies of cellular senescence within hepatic tissue [6, 9, 10]. In a pioneering endeavour, the research team led by Yang et al. has charted the first‐ever single‐cell nuclear transcriptome atlas specific to primate liver aging [11]. This comprehensive atlas highlights key characteristics of primate liver aging, including lipid accumulation, heightened fibrosis, immune cell infiltration and metabolic disruption. Additionally, the study posits that increased expression of the transcription factor SREBP2, associated with cholesterol synthesis, acts as a driving force behind hepatocyte senescence. These findings not only establish a theoretical foundation for the development of prognostic and interventional strategies for liver aging and its related diseases but also underscore the multifaceted nature of liver aging, influenced by both intrinsic cellular aging within hepatic parenchymal and non‐parenchymal cells and the dynamic interplay of the cellular microenvironment in the aging process [11, 12].

The extracellular matrix (ECM) has been identified as the thirteenth aging marker [13]. The extracellular matrix (ECM), comprised of components such as proteoglycans, collagens and elastic fibres, is a crucial constituent of the microenvironment [14, 15]. Although the precise mechanisms governing the ECM's role in liver aging remain elusive, existing research suggests its involvement in facilitating tumour cell support, adhesion, migration and invasion during metastasis [16]. Furthermore, ECM over‐deposition and remodelling are pivotal factors in conditions like liver fibrosis and pulmonary fibrosis [17, 18]. In arthritis and joint cartilage degeneration, ECM degradation and weakened structural support contribute to joint damage and pain [19]. Additionally, cardiac ECM remodelling is linked to cardiovascular ailments such as myocardial hypertrophy, heart failure and atherosclerosis [20, 21]. Our previous studies have also indicated that specific ECM components can enhance the functional maturation of liver cells [22]. Investigating the crosstalk between the ECM and liver cells during the aging process sheds light on the declining regenerative capacity of the liver and overall physiological function. Moreover, it provides an opportunity to develop innovative strategies for rejuvenation, potentially delaying the aging process and preventing liver function failure [23].

In this study, quantitative proteomics techniques were employed to systematically characterise the alterations in the extracellular matrix of the liver throughout its entire lifecycle, from neonatal stages to senescence. Specifically, our findings underscore the pivotal role of glycosaminoglycan molecules in facilitating tissue development and mediating signal transduction pathways. Most notably, our research demonstrates that the application of the glycosaminoglycan Lumican significantly sustains the stemness of hepatic progenitor cells and enhances hepatocyte proliferation. This discovery proposes a novel strategic approach for advancing studies in hepatocyte culture and liver regeneration.

## 2 Results

### 2.1 Liver Matrisome Profile of Different Ages

The liver exhibits varying functional trends across different age groups, including differences in cellular proliferation capacity, albumin synthesis and drug metabolism. For instance, the proliferation marker Ki67 is prominently expressed in the liver at 1 week of age, while ALB expression reaches its peak at 8 weeks in adult mice but declines in elderly rats (Figure S1A). Similarly, AFP, a marker indicative of stemness, exhibits its highest expression levels in the livers of 1‐week‐old rats, whereas key functional markers like urea synthesis gene CPS1 and drug metabolism gene CYP1A2 are most prominently expressed in mature rat livers (Figure S1B).

**FIGURE 1:**
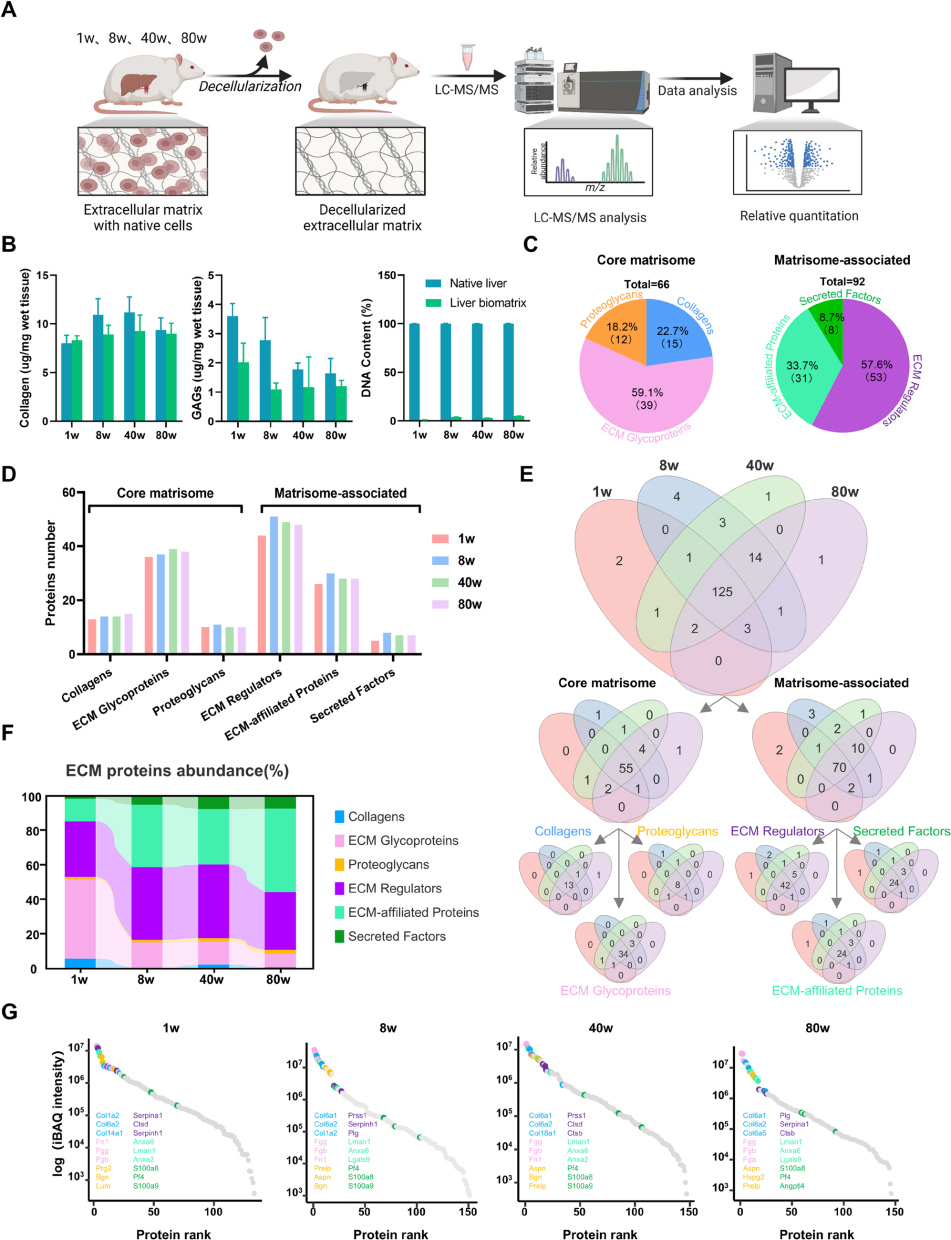
**ECM** Profiles in Rat Liver Across Various Ages. (A) Decellularisation and proteomic workflow for rat liver. This schematic illustrates the process of decellularisation and subsequent proteomic analysis conducted on liver samples from rats at 1, 8, 40 and 80 weeks. (B) Quantitative analysis of key ECM components such as collagens, glycosaminoglycans (GAGs), and DNA in decellularised liver scaffolds from rats at various ages. (C) Distribution of ECM protein categories. (D) The counts of ECM proteins in liver scaffolds from rats at different ages, illustrating age‐related changes in ECM composition. (E) A Venn diagram representing the overlap of ECM protein expression in decellularised liver scaffolds across different age groups. (F) Bar charts depict the proportion of various ECM types present in liver scaffolds from four developmental stages. (G) The abundance of ECM proteins in decellularised liver scaffolds from rats aged 1, 8, 40 and 80 weeks, highlighting key proteins at each stage.

To investigate the role of the liver's extracellular matrix (ECM) in modulating these functions, we employed our previously established decellularisation method to obtain decellularised liver scaffolds [24] (Figure 1A). Through a gentle and efficient four‐step decellularisation process via in situ perfusion, we obtained liver decellularised scaffolds that retained a significant amount of collagens (Figure 1B), glycosaminoglycans (GAGs) and major protein components (Figure S2A). HE staining, along with DAPI staining, confirmed the nearly complete removal of cellular elements (Figure S2B,C).

Quantitative proteomics constitutes a critical method for identifying ECM components secreted into and situated within the extracellular space. To elucidate the molecular mechanisms underlying liver aging, we utilised quantitative proteomics to examine the composition of the ECM in decellularised biomatrices derived from livers of various ages. Utilising this dataset, we constructed a detailed map of the liver ECM, revealing developmental changes in the ECM spanning from youth to old age. Pearson correlation coefficients demonstrated a high positive correlation among the groups (correlation coefficients: 0.8–0.943) (Figure S3). According to annotations from MatrisomeDB 2.0, ECM proteins were classified into “core matrisome” proteins, including collagens, glycoproteins and proteoglycans and “matrisome‐associated” proteins, encompassing ECM‐related proteins, ECM regulators and secretory factors [25, 26]. Our study identified 158 ECM proteins spanning six categories (Figure 1C), with 45, 46, 29 and 38 highly expressed ECM proteins detected in liver biomatrices at 1w, 8w, 40w and 80w, respectively (Figure 1D and Figure S4). Across all four age stages, we observed 125 common ECM proteins, representing an overlap rate of approximately 80% (Figure 1E). The greatest overlap occurred in the category of matrisome‐associated proteins, with ECM regulators exhibiting the highest unique proportion at 8 weeks.

In terms of protein abundance, glycoproteins were observed to be the most abundant across different ages, correlated with the liver's robust synthesis, secretion and metabolic functions [27, 28]. The expression levels of identified secretory factors were markedly lower in older rats compared to the other three age groups (Figure 1F), potentially indicative of a decline in liver function associated with aging. An analysis of ECM protein distribution across age stages revealed that ECM glycoproteins were predominantly expressed, with fibronectin and fibrinogen consistently exhibiting high expression across all age groups (Figure 1G). As rats aged, the expression of various collagens types in the liver exhibited changes. In the early stages (such as 1w and 8w), high expression of Col1a2 and Col6a2 may contribute to the development of liver tissue stability and the formation of the extracellular matrix [29, 30, 31]. Over time, at 40w and 80w, the elevated expression of Col6a1 and Col6a2 could be associated with liver fibrosis and extracellular matrix regulation [32, 33]. Additionally, the high expression of Col18a1 may also be involved in processes such as angiogenesis, tissue repair and liver function recovery [34, 35]. Overall, the variations in collagens are closely linked to tissue remodelling, fibrosis and the stability of the extracellular matrix during liver aging. Generally, core matrisome proteins, playing a supportive role, continue to constitute a major component of the ECM composition. However, with advancing age, the changes in ECM‐associated proteins become more pronounced and may assume a dominant regulatory role.

### 2.2 Temporal Expression Characteristics of the Hepatic Extracellular Matrix

**FIGURE 2:**
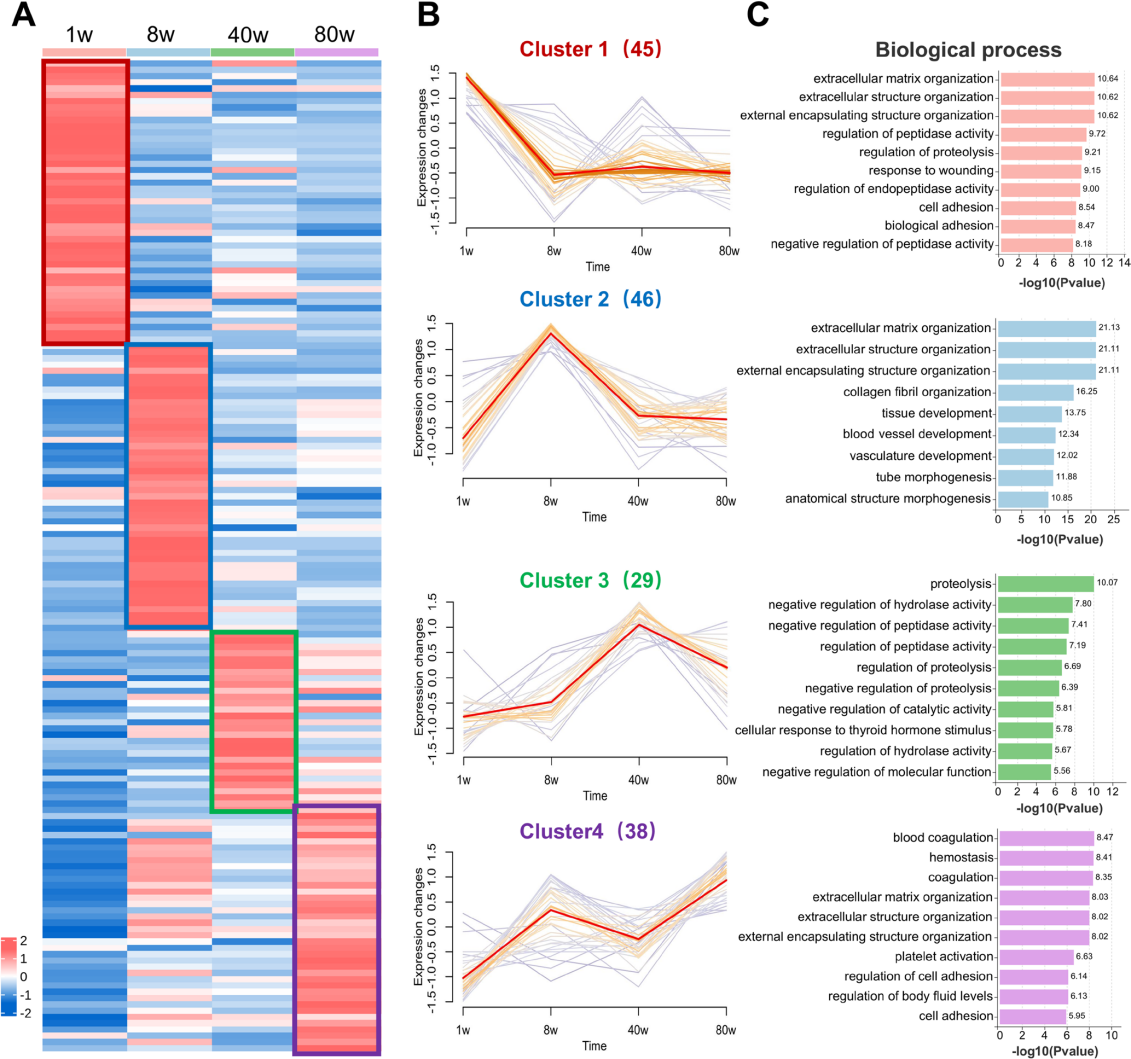
Age‐dependent expression patterns and biological processes of liver ECM proteins. (A) Heatmap of the temporal expression trends for ECM proteins across different age groups. (B) Four distinct clusters (Cluster 1 to Cluster 4) are identified, each associated with specific biological functions and developmental stages of the liver. (C) Detailed analysis of the biological processes enriched in each cluster, demonstrating the functional shift in the liver's ECM as it ages.

Figure 2A displays a heat map illustrating the variations in protein abundance detected within the decellularised liver scaffolds of rats across four different age groups. Hierarchical clustering analysis has identified four distinct clusters, designated as Clusters I, II, III and IV, reflecting the trends in the major components within the liver scaffolds at various developmental stages. Additionally, a line graph (Figure 2B) dynamically displays these changes over time, highlighting the temporal progression of protein expression levels.

Further analysis via Gene Ontology (GO) enrichment of these clusters reveals distinct functional profiles at different stages of liver development (Figure 2C). Cluster I, corresponding to 1‐week‐old rats, highlights proteins essential for early liver development. These proteins play pivotal roles in cellular biosynthesis, hepatocyte proliferation and the development of the neonatal rat liver [22]. The regulation of protease and endopeptidase activities within this cluster underscores the critical role in shaping the hepatic microenvironment during early life stages. At 8 weeks, the protein composition of Cluster II within the liver scaffolds correlates with extracellular matrix architecture, tissue development and angiogenesis [36]. These processes are crucial for the metabolic functions of the adult rat liver. By 40 weeks, Cluster III demonstrates a shift in the liver scaffold's protein composition towards protein hydrolysis, enzymatic activity regulation and molecular function modulation. This cluster reflects the liver's need to regulate enzymatic activities and molecular functions during mid‐life, likely as a mechanism to manage tissue remodelling and protein turnover. The negative regulation of protein hydrolysis suggests a balancing mechanism to mitigate excessive degradation, thereby maintaining the structural and functional integrity of the ECM [37]. In the oldest age group, Cluster IV of the liver scaffold's ECM composition is markedly influenced by proteins associated with coagulation, haemostasis and platelet activation. This cluster highlights components related to scaffolds prepared from 80‐week‐old rats, potentially linked to the liver's coagulation, haemostasis and fluid regulation processes at an advanced age [38].

The clusters identified through this analysis distinctly delineate the temporal progression of liver ECM composition, reflecting the organ's functional evolution from neonatal development through to senescence. The early stages are characterised by growth and development, while maturity is marked by a robust ECM, which supports the liver's metabolic demands. The middle age phase reveals a focus on the regulation of protease balance, and in advanced age, the ECM increasingly involves processes related to haemostasis. These age‐related transformations in the liver ECM underscore the liver's complex and dynamic role in systemic physiology and its gradual transition towards reduced functional reserve with aging. This narrative not only elucidates the progressive changes in liver functionality but also highlights the liver's adaptability in response to physiological demands over the lifespan.

### 2.3 Age‐Related Dynamics of Liver ECM Molecules and Receptor Interactions

**FIGURE 3:**
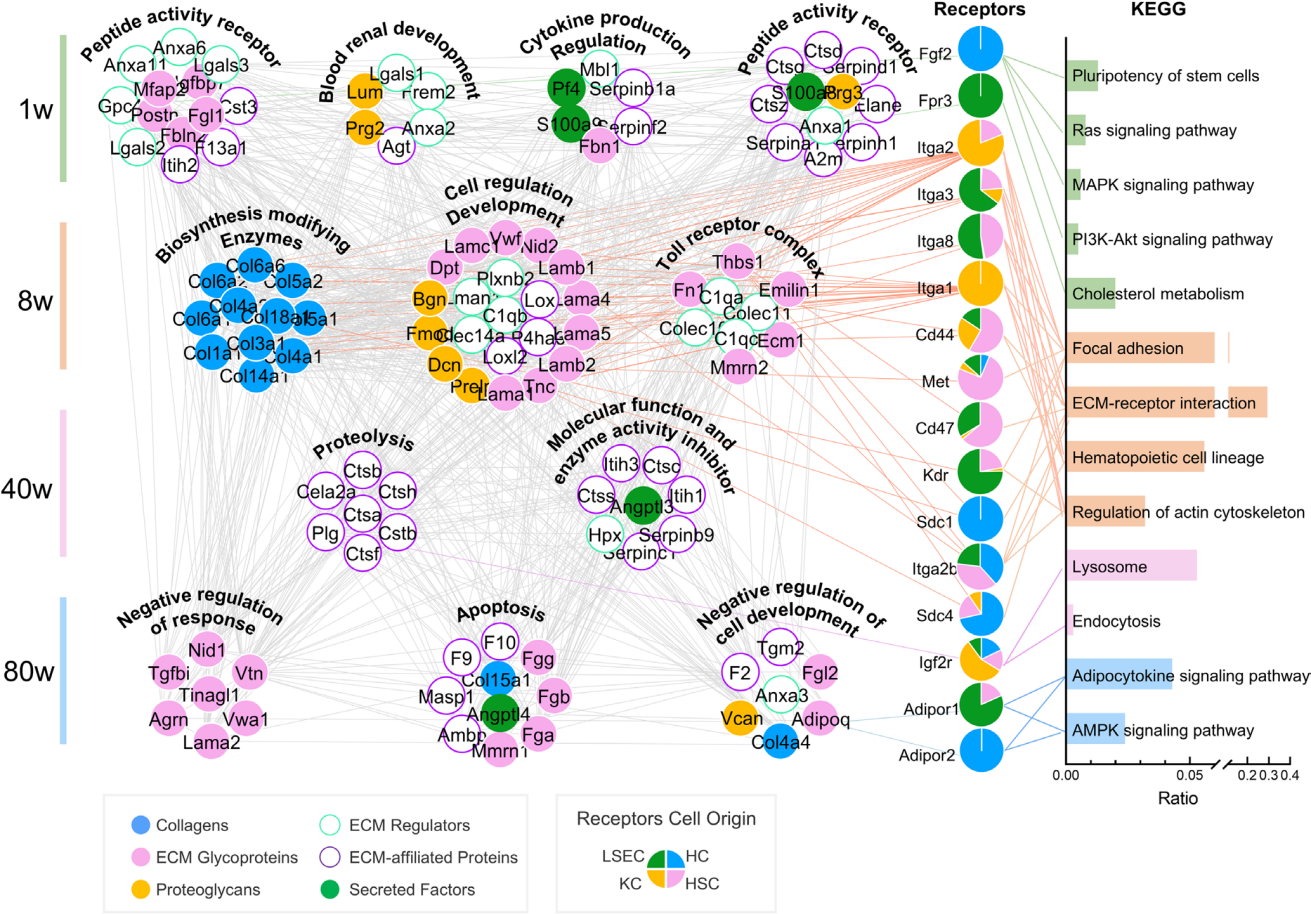
Comprehensive analysis of age‐specific liver ECM molecules and receptor‐mediated signalling pathways in rats. Left, the interactions between soluble factors and core matrix components essential for liver development. These interactions are crucial for the early establishment of liver structure and function. The complex network of interactions between hepatic cell receptors and ECM molecules is described. Right, KEGG analysis reveals potential signalling pathways triggered by receptor‐ECM interactions.

The ECM, a pivotal component of the cellular microenvironment, plays a critical role in regulating cellular functions through its specific molecular interactions with surface receptors on cells, thereby activating intracellular downstream signalling pathways [39]. Our comprehensive analysis and comparison of ECM molecular expression patterns and their receptor interactions in rat livers at different developmental stages have elucidated a finely tuned, age‐specific molecular regulatory network (Figure 3). Initially, we identified ligand‐receptor molecules associated with the 158 enriched ECM components by integrating proteomic data from the entire rat liver with existing ligand‐receptor databases [40]. Coupled with STRING database analysis, we identified 16 receptors interacting with these ECM ligands. Utilising cell type‐specific liver proteome mapping data from He et al. [41], we annotated the likely cellular origins of these receptors. As depicted in Figure 3, colour‐coded regions within the central pie chart delineate the proportional cell sources of the relevant molecules: HC for hepatocytes, KC for kupffer cells, LSEC for hepatic sinusoidal endothelial cells and HSC for hepatic stellate cells. Through KEGG enrichment analysis, we evaluated the interactions of key receptors, such as ITGA1 and ITGA2, with signalling pathways, including those related to stem cell pluripotency and the interplay between the Ras signalling pathway and ECM‐receptor interactions.

In the liver of 1‐week‐old neonatal rats, biological processes involving the primary expression of regeneration‐functional receptors in the extracellular matrix (ECM) are critical for regulating stem cell pluripotency, as well as cell proliferation and development. Notably, during the neonatal period, the liver maintains robust regenerative capacity. The FGF2 receptor‐mediated pathway actively supports stem cell pluripotency—the potential of stem cells to differentiate into all three germ layers (endoderm, mesoderm, ectoderm), a capability shared by embryonic stem cells (ESCs) and induced pluripotent stem cells (iPSCs) [42, 43, 44]. FGF2, a key factor in maintaining stem cell pluripotency, plays a critical role in driving rapid hepatocyte proliferation and organ development by activating signalling pathways such as ERK and PI3K‐AKT/mTOR [45, 46]. At this stage, regulation of the cholesterol metabolic pathway involving the Fpr3 receptor is essential for initiating lipid metabolic functions [47]. As the liver enters the 8‐week adolescent stage, the expression of extracellular matrix (ECM) molecules is closely associated with hepatic structural remodelling activities, including cell regulation, ECM interactions, regulation of haematopoietic lineages and cytoskeletal modulation. Receptors such as Cd44, Cd47, SDC1, SDC4 and members of the integrin family are involved in regulating ECM‐receptor interactions and focal adhesion, marking the initiation of hepatic structural remodelling and promoting the formation of hepatic lobules [48, 49, 50]. Concurrently, their involvement in the regulation of haematopoietic cell lineages signifies the transition of liver haematopoietic function from the neonatal to adult state [51, 52]. Regulation of the actin cytoskeleton, involving proteins such as Kdr and Met, indicates the establishment of cell polarity and the formation of the biliary network [49]. Additionally, activation of Met is critical for cell growth and migration [53, 54]. In 40‐week‐old mature rats, ECM molecules are more closely associated with metabolic activity functions. As the primary clearance receptor for IGF2, the IGF2R receptor enhances degradation and recycling capabilities. By internalising and degrading IGF2, it maintains the concentration balance of IGF2 in the tissue microenvironment [55, 56], playing a key role in determining cell fate and tissue regeneration [57, 58]. At 80 weeks of age, the expression of hepatic ECM molecules exhibits a compensatory tendency to regulate cellular metabolic processes. Receptors such as Adipor1 and Adipor2 may be involved in adiponectin signalling pathways and cytokine secretion dysregulation associated with aging‐related adipose tissue, influencing liver metabolism. Both receptors participate in the AMPK signalling pathway, which is closely linked to the regulation of cellular stress responses and energy metabolism [59, 60, 61] (Figure 3).

### 2.4 Regulatory Pattern of ECM in the Process of Liver Aging

**FIGURE 4:**
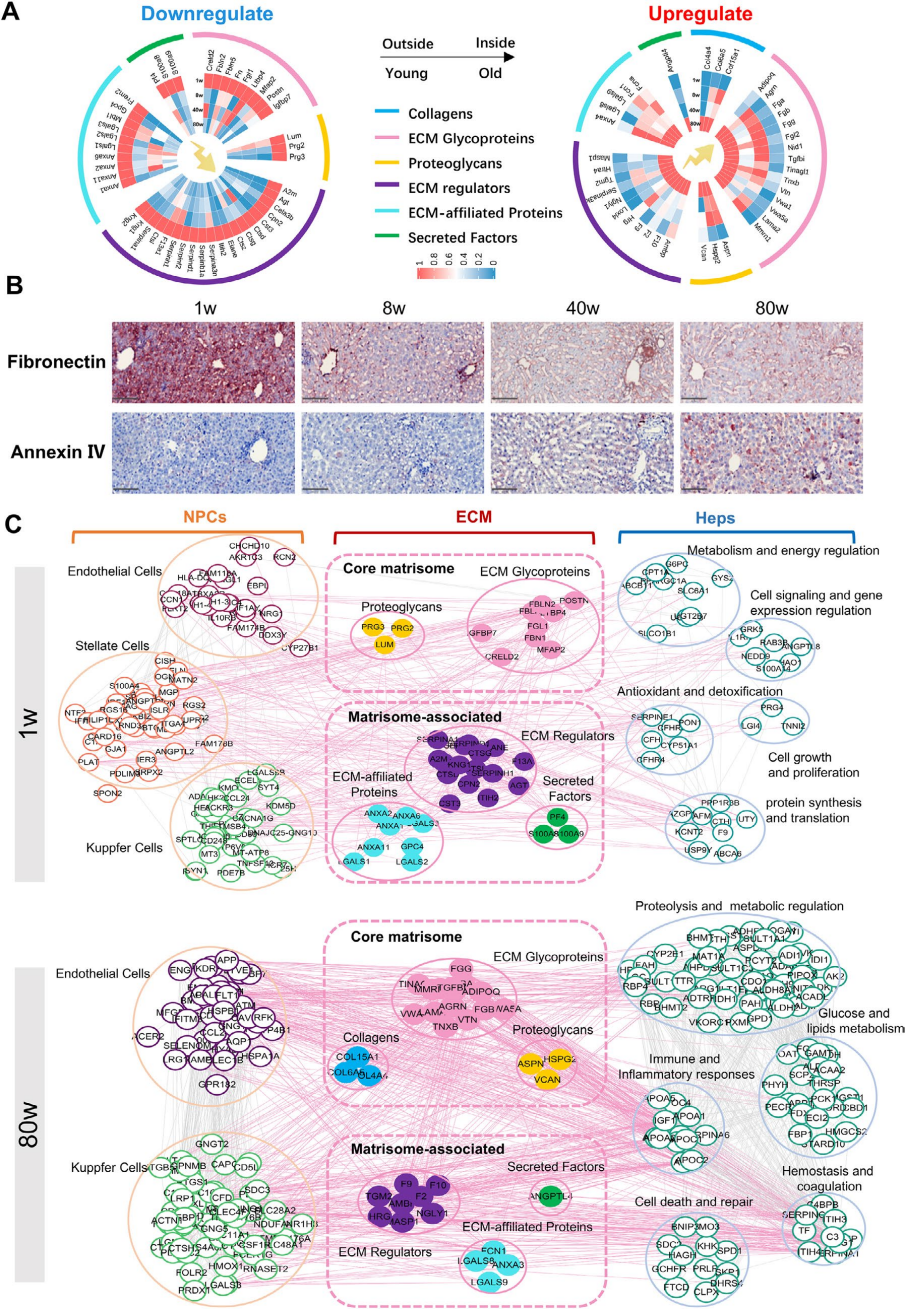
Interaction networks of specific high expression ECM molecules with parenchymal and non‐parenchymal cells in childhood and old age. (A) Gradual increase and decrease of ECM proteins from early childhood to old age. (B) Immunohistochemical staining results of Fibronectin and Annexin IV in liver at four different age stages. Scale bar =100 μm. The experiment was run in triplicate. (C) Network of interactions between ECM molecules with parenchymal and non‐parenchymal cells with high expression in the neonatal stage (1w) and old age (80w).

Here, we focus on ECM proteins that gradually increase or decrease from young children to old age. At present, cellular markers of neonatal liver development and liver aging have been well studied, while ECM markers have not been well studied. In four age groups, we found that 45 ECM proteins were down‐regulated with age and 38 ECM proteins were up‐regulated with age (Figure 4A). Our results suggest that during liver development, ECM expression of Fbn1, Fn, Ltbp4, Serpinf2, Ctsd, Prg2, etc., which are important components of the extracellular matrix, may play a regulatory role in liver development and are essential for liver cell development and functional maintenance, decreasing gradually [36, 62, 63]. Matrix protein molecules such as Adipoq, Angptl4, Col4a4, Hrg, Tgfbi, Tgm2, Vcan and Vtn increase with age, and they may promote the occurrence of fatty liver during aging by affecting fat accumulation and metabolic disorders [64, 65]. By affecting inflammation and repair mechanisms in the liver, it is associated with oxidative stress and aging in the liver and plays a role in cell adhesion and migration, potentially affecting the liver's ability to regenerate and repair [66, 67, 68]. We selected Fn (Fibronectin) and Anxa4 (Annexin IV) for immunohistochemical staining to verify the expression patterns during aging (Figure 4B). The expression of Fibronectin is highest at 1w, and it is expressed in many kinds of cells, such as hepatic sinusoidal endothelial cells and hepatic parenchymal cells. With the increase of age, the expression of fibronectin decreases significantly. In contrast, Annexin IV is highly expressed at 80w and its expression increases with age.

We integrated and analysed the ECM molecules that are highly expressed in neonatal and old age with the published single‐cell data on neonatal liver development [69] and liver aging [70], respectively, and found that the ECM molecules that are highly expressed in the neonatal stage are correlated with endothelial cells, stellate cells and the liver microenvironment. Non‐parenchymal cells such as Kupffer cells form a close interaction network with multiple molecules of parenchymal cells. These molecules are involved in metabolism and energy regulation, cell signalling and gene expression regulation, antioxidant and detoxification, cell growth and proliferation, protein synthesis and translation, and other functions related to liver development in hepatocytes. The 38 ECM molecules that are highly expressed in old age also interact with multiple molecules from endothelial cells, stellate cells, Kupffer cells and liver parenchymal cells. They are also involved in functional processes related to protein hydrolysis and metabolic regulation, glucose and lipid metabolism, immune and inflammatory responses, cell death and repair, haemostasis and clotting, related to liver aging (Figure 4C).

### 2.5 Proteoglycan Dynamics Within the Liver ECM and Developmental Regulation

The majority of matrisome‐associated components, including growth factors, chemokines and cytokines, serve as regulatory agents by interacting with the core matrisome, thereby facilitating cellular functions [71, 72]. In this study, we identified 92 matrisome‐associated proteins within our decellularised liver scaffold. Theoretically, these hydrophilic secretory factors are highly susceptible to loss during the decellularisation process unless they are covalently bonded to the ECM structure. The retention of these factors suggests their integral roles within the ECM framework. Proteoglycans, a class of multifunctional glycoproteins within the ECM, are characterised by their extensive polysaccharide side chains and a core protein matrix [73]. These molecules not only bind to and store bioactive signalling molecules through their sulfated glycosaminoglycan (GAG) carbohydrate chains but also regulate the release of these signalling molecules, playing a pivotal role in cellular communication [74]. The epitopes of GAG chains form complexes with soluble signalling molecules, and through synergistic interactions with specific receptors, activate intracellular signalling pathways [75]. This mechanism is crucial for regulating cellular behaviours, including proliferation, differentiation and migration.

**FIGURE 5:**
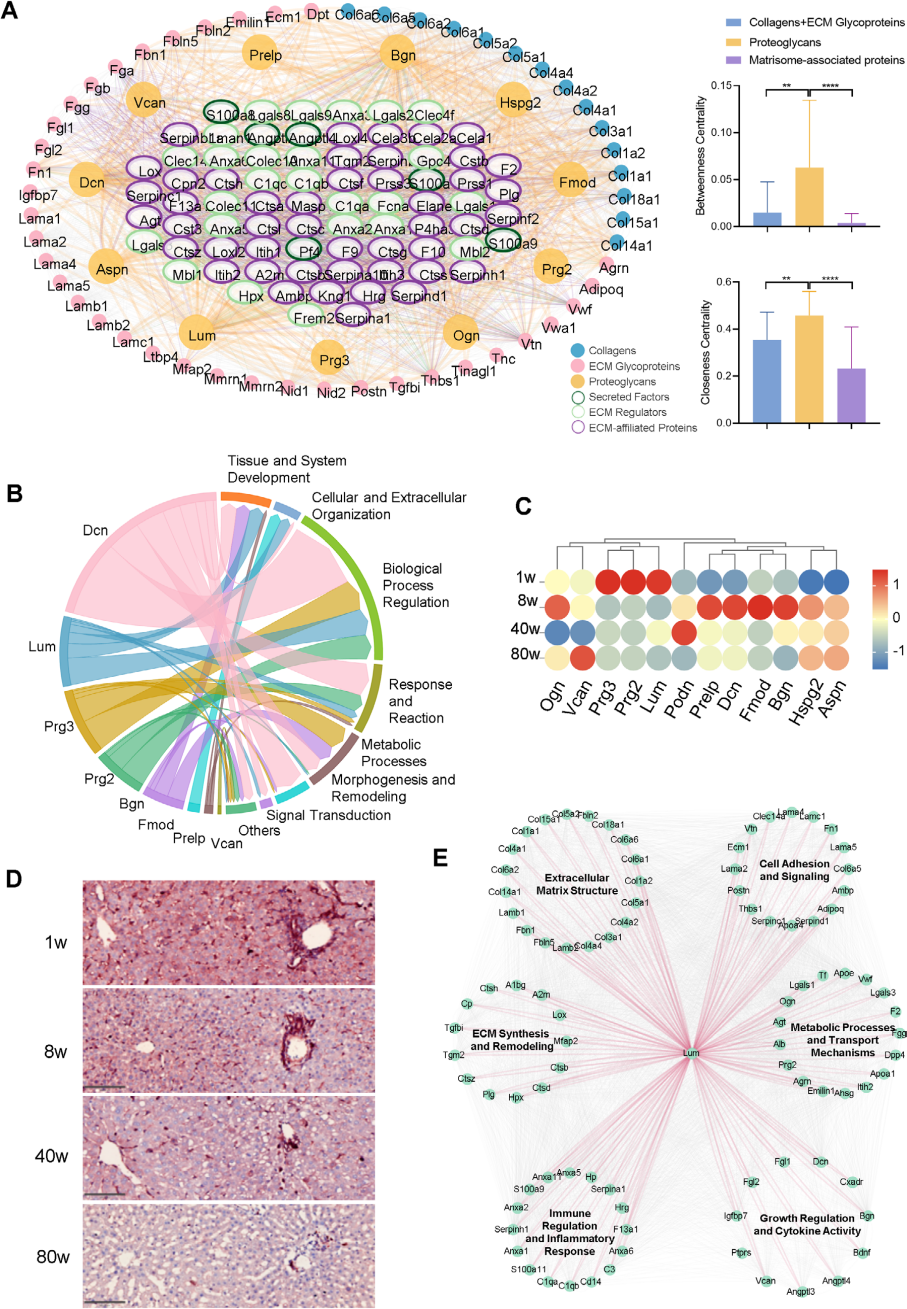
Proteoglycan dynamics within the liver ECM and developmental regulation. (A) A complex network of interactions between proteoglycans and a large number of stroma‐associated proteins. Using Cytoscape software, Force Atlas2 is used to lay out the visualisation network and classify node colours based on matrix proteome gene annotations (e.g., proteoglycans labelled orange). The line indicates the protein interaction recorded in the STRING database, and its thickness indicates the confidence level of the interaction. (B) The multiple biological functions of proteoglycans identified in the liver. (C) The expression patterns of identified proteoglycans in the liver at different developmental stages. (D) Immunohistochemical staining results of Lumican in rat livers at different developmental stages. Scale bar = 100 μm. The experiment was run in triplicate. (E) Lumican interacts with a variety of matrix protein and cytokine analysis networks, as well as key biological processes involved in liver development.

Utilising the STRING database and Cytoscape Network Analyser module, we conducted an analysis of 158 ECM interactions and enriched topological analysis. We elucidated the complex interaction network among collagens, ECM glycoproteins, proteoglycans and numerous matrisome‐associated proteins. These interactions encompass various proteoglycans, including Htra4 (high‐temperature requirement protein A4), Plg (plasminogen), C1qb (complement component 1q subunit B), serpinf2 (serine protease inhibitor family F member 2, also known as pigment epithelium‐derived factor, PEDF), Ctsa (cathepsin A), Prss3 (protease serine 3), Anxa2 and Anxa5 (annexin A2 and A5), Lgals3 (galectin 3), S100a8 and S100a9 (S100 protein family members), Serpina1 (α‐1 antitrypsin), Loxl2 and Loxl4 (lysyl oxidase‐like 2 and 4), Itih1 and Itih3 (α‐1 antitrypsin inhibitor heavy chain 1 and 3), Serpina10 (α‐1 antithrombin), Angptl3 and Angptl4 (angiopoietin‐like protein 3 and 4) (Figure 5A). Through the complex network parameters analysis of each node in the network using the Cytoscape Network Analyser module, proteoglycans showed higher betweenness centrality than collagens, ECM glycoproteins and numerous matrisome‐associated proteins (including secreted factors, ECM regulators and ECM‐affiliated proteins), indicating that proteoglycans exhibit stronger interactions with other ECM proteins. Additionally, the closeness centrality of the proteoglycans molecule group was significantly higher than that of collagens, ECM glycoproteins and numerous matrisome‐associated proteins, demonstrating that proteoglycans occupy a more central position within the entire interaction network, linking scaffold proteins such as collagens and ECM glycoproteins with matrisome‐associated proteins (Figure 5A). Moreover, our results cover the diverse biological functions these proteins serve in the liver, particularly their critical regulatory roles in angiogenesis, tissue repair, immune modulation and cellular signal transduction (Figure 5B). These findings underscore the multifunctionality of proteoglycans within the liver ECM. They not only interact with matrisome‐associated components but also associate further with ECM scaffold proteins, such as collagens and glycoproteins. These interactions not only enrich the composition of the liver matrix but also constitute essential components of the cellular microenvironment, crucial for maintaining the structural integrity and functionality of the liver.

Further analyses of the biological processes involving the aforementioned proteoglycans reveal that decorin (Dcn) participates in a broad spectrum of biological processes, including tissue development, organisation of cells and extracellular structures, regulation of biological processes, responses and reactions, as well as metabolic processes. Lumican (Lum) and Pregnancy‐Specific β‐1‐Glycoprotein 3 (Prg3) also demonstrate involvement in various biological processes (Figure 5B). In summary, core‐matrisome proteoglycans serve as principal reservoirs for soluble and bioactive factors within the liver tissue microenvironment, playing a pivotal role in facilitating their functional mechanisms within the tissue.

Subsequent analyses of the identified proteoglycans revealed distinct expression patterns in the liver across different developmental stages. Notably, in one‐week‐old neonatal rats, Lum, Prg2 and Prg3 demonstrated significantly enriched expression (Figure 5C). Given their regulatory roles and biological functions, it is hypothesised that Lumican may play a crucial role in the development of the neonatal liver. Immunohistochemical staining confirmed the elevated expression of Lumican in the liver at one week, with a gradual decrease observed as age increased (Figure 5D). Protein interaction analyses indicated that Lumican engages in multiple interactions with various matrix proteins and cytokines, participating in the regulation of critical biological processes related to liver development, such as ECM Structure, ECM synthesis and remodelling, Immune regulation and inflammatory response, Cell adhesion and signalling, Metabolic processes and transport mechanisms, Growth regulation and cytokine activity (Figure 5E). These findings provide new insights into the complex regulatory networks of the ECM during liver development and in disease states.

### 2.6 Lumican‐Mediated Regulation of Hepatic Stemness and Proliferative Capacity During Liver Development and Maturation

During the developmental process, the liver exhibits a progressive decline in its cellular stemness and proliferative capacity [76]. In mature livers, the capacity for hepatocyte self‐renewal is relatively limited, especially evident in the absence of external disturbances in adult states [77, 78]. Our study, employing qRT‐PCR analysis of neonatal and adult rat liver cells, revealed that the expression level of Lumican is significantly higher in the neonatal liver compared to that in the adult liver (Figure S5A). Immunofluorescence staining further corroborated these findings: Lumican and PCNA demonstrate reduced expression levels in the adult liver, whereas both exhibit high expression in the neonatal liver, with numerous instances of co‐expression observed. These findings suggest that Lumican is expressed at elevated levels in neonatal liver tissues, which demonstrate stem cell‐like properties (Figure S5A).

**FIGURE 6:**
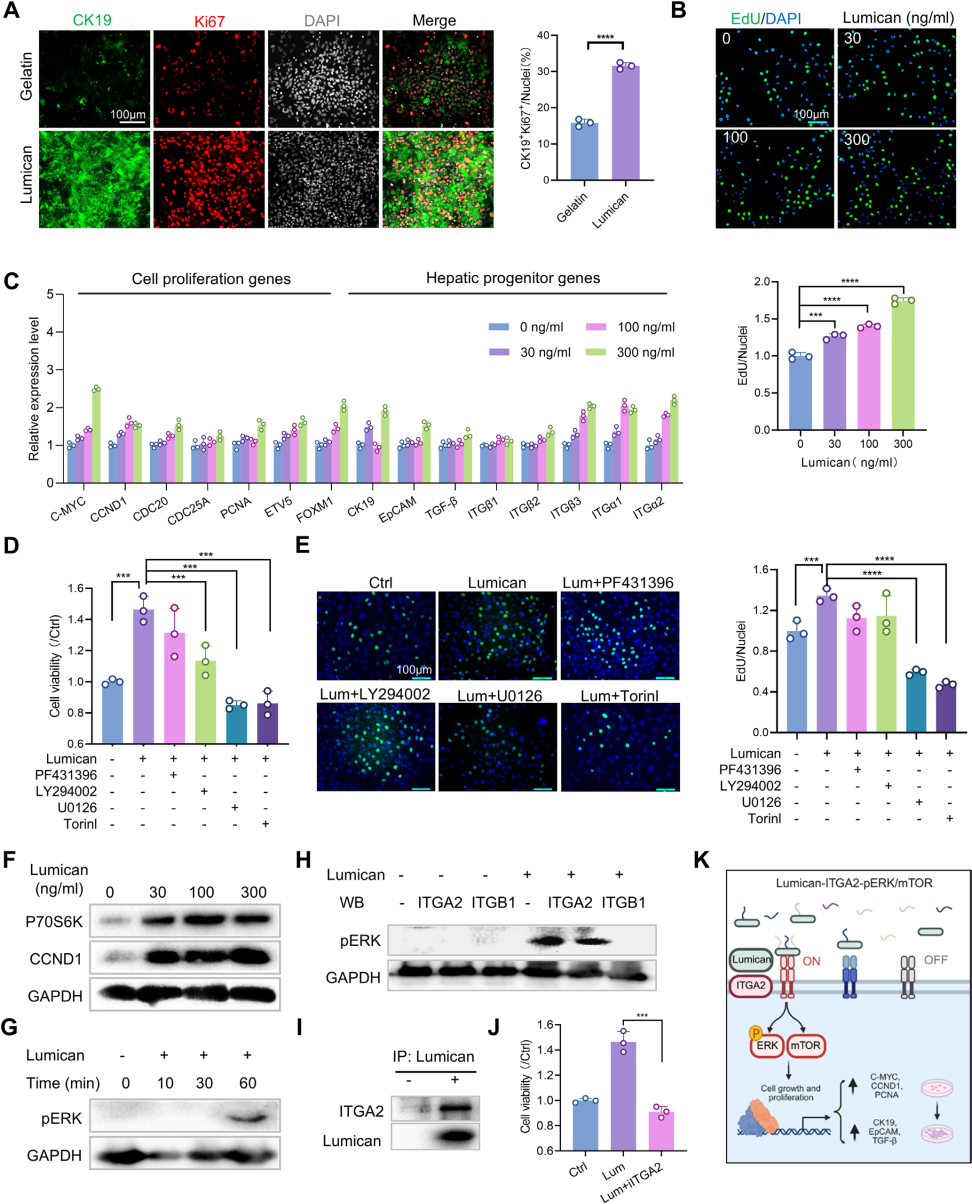
Lumican binds to ITGA2 and activates ERK and mTOR signalling pathways to participate in the regulation of cell proliferation. (A) Immunofluorescence results of CK19 and Ki67 in neonatal hepatocytes co‐cultured with Lumican (30 ng/mL). Scale bar =100 μm. (B) EdU staining results of HepaRG cells treated with different Lumican concentrations for 12 h. Scale bar =100 μm. (C) With the increase of Lumican concentration, RNA expression levels of genes related to cell proliferation and stem cell property and functional genes of mature hepatocytes changed. (D) Cell viability results of Lumican‐treated HepaRG cells with the addition of several common proliferation signalling pathway inhibitors, PF431396 (a FAK inhibitor), LY294002 (a PI3K inhibitor), U0126 (an inhibitor of pERK) and Torin1 (an mTOR inhibitor). (E) EdU staining results and statistical analysis results of HepaRG cells treated with several common proliferation signalling pathway inhibitors and Lumican (30 ng/mL). Scale bar =100 μm. (F) The expression levels of proteins downstream of the ERK pathway and mTOR pathway changed in HepaRG cells treated with Lumican at different concentrations for 12 h. (G) The expression level of pERK protein, an activation marker of the ERK pathway, changed in HepaRG cells after Lumican treatment at 30 ng/mL for different times. (H‐I) Results of co‐immunoprecipitation of ITGA2 with pERK or Lumican (30 ng/mL) in HepaRG cells. (J) Experimental results of cell proliferation in HepaRG cells treated with the ITGA2 inhibitor E7820. (K) Schematic illustration of Lumican regulating cell proliferation by binding to ITGA2, thereby activating the ERK and mTOR signalling pathways. (A), (B), (D), (E), (J), ***, *p* ≤ 0.001; ****, *p* ≤ 0.0001; two‐tailed Student's t‐tests. All experiments were run in triplicate.

A detailed analysis revealed that Lumican is extensively expressed across various hepatic cell subtypes in the neonatal liver, while its expression is notably reduced in the adult liver due to the lower proliferative capacity of the parenchymal cells. In contrast, hepatic stellate cells and endothelial cells, which retain some proliferative ability, exhibit relatively higher levels of Lumican expression (Figure S5B). This suggests a close correlation between Lumican expression and the proliferative capacity of cells. When neonatal liver cells were co‐cultured with Lumican, immunofluorescence staining revealed that the proportion of CK19 and Ki67 double‐positive cells in the Lumican‐treated group was significantly higher than in the control group by the fourth day (Figure 6A), demonstrating that Lumican significantly promotes the proliferation of neonatal liver cells. Additionally, as the concentration of Lumican increased, there was a significant enhancement in the fluorescence intensity of liver stem cell markers SOX9 and AFP, while the expression of mature liver cell markers such as CPS1, ALB and CYP3A41A correspondingly decreased (Figure S6A,B). This further confirms the role of Lumican in maintaining cellular stemness.

To investigate the potential mechanisms underlying Lumican's effects, we utilised the human hepatic progenitor cell model HepaRG for our experiments. HepaRG cells exhibit characteristics of hepatic progenitor cells, including cellular phenotypes and gene expression patterns [79, 80]. Under varying concentrations of Lumican, the proliferative capacity of HepaRG cells exhibited significant concentration‐dependent variations (Figure S7A). EdU staining results indicated that the percentage of cells in the S phase increased synchronously with rising Lumican concentrations (Figure 6B). qRT‐PCR analysis in Figure 6C also showed that the expression levels of genes associated with cell proliferation and stemness, such as C‐MYC, CCND1, PCNA, CK19 and EpCAM were upregulated with increasing concentrations of Lumican. Immunofluorescence staining was performed on HepaRG cells treated with Lumican after starvation treatment (Figure S7B). It was found that under the same exposure time, the fluorescence of common stemness markers SOX9 and AFP in Lumican treatment group was stronger than that in control group. Through the determination of the functional activity of CYP3A4 and the synthetic function of ALB, it was found that compared with the control group, both of these two indicators in the Lumican treatment group showed reductions to varying degrees, and there were statistically significant differences. Lumican can delay the hepatic maturation of HepaRG cells (Figure S8). The Lumican gene in HepaRG cells was silenced by siRNA. The high‐efficiency siRNA was screened by the CCK8 method. The HepaRG cells treated with this siRNA were set as the siLum group for qRT‐PCR detection, and it was found that the expression level of Lumican in the siLum group was lower than that in the control group. The results confirmed that the siRNA we screened could efficiently silence the Lumican gene and inhibit the proliferation of HepaRG cells (Figure S9).

Currently, several well‐known proliferative signalling pathways include the FAK, PI3K, ERK and mTOR pathways [81, 82]. We cultured Lumican‐treated HepaRG cells in the presence of inhibitors for each of these signalling pathways. Both cell proliferation assays and EdU staining results indicated that, compared to PF431396 (a FAK inhibitor) and LY294002 (a PI3K inhibitor), U0126 (an inhibitor of pERK) and Torin1 (an mTOR inhibitor) significantly inhibited cell proliferation, a statistically significant finding (Figure 6D,E). Moreover, there were no significant differences between these two groups of inhibitor‐treated cells and the control group, suggesting that the ERK and mTOR pathways may be the signalling pathways through which Lumican exerts its effects. In HepaRG cells treated with varying concentrations of Lumican for 12 h, there was an increase in the expression of proteins downstream of the ERK pathway, such as CCND1, and downstream of the mTOR pathway, such as P70S6 kinase (P70S6K), correlating with increasing concentrations of Lumican (Figure 6F). Furthermore, the activation marker of the ERK pathway, pERK, was expressed as early as 60 min after treatment (Figure 6G).

Previous research indicates that ITGA2 and ITGB1 may serve as ligands through which Lumican exerts its effects [83, 84]. Co‐IP experiments have revealed the presence of pERK in the ITGA2 group (Figure 6H), along with an interaction between ITGA2 and Lumican (Figure 6I). When HepaRG cells are treated with the ITGA2 inhibitor E7820, a significant reduction in cell proliferation is observed compared to cells treated solely with Lumican (Figure 6J). Therefore, we hypothesise that Lumican may regulate cell proliferation by binding to ITGA2, thereby activating the ERK and mTOR signalling pathways (Figure 6K). In summary, Lumican likely plays a crucial role in neonatal liver development and liver regeneration, modulating hepatocyte proliferation and the maintenance of stemness through specific signalling pathways and receptor interactions. These findings not only provide new insights into the molecular mechanisms underlying liver development and regeneration but also offer potential therapeutic targets for future liver disease treatments.

## 3 Discussion

The liver, as the largest digestive gland and parenchymal organ in the human body, performs critical functions including detoxification, metabolism, bile secretion, synthesis of secretory proteins and immune defense [5]. This study delves into the dynamic changes of the ECM in the liver during processes of liver development, regeneration and aging, and examines its impact on hepatic function, thereby elucidating the pivotal role of ECM in hepatic development and aging. The liver ECM comprises core matrisome components and matrisome‐associated regulatory factors. We identified a total of 158 matrix protein molecules, which collectively form a complex three‐dimensional network that profoundly influences cellular processes such as proliferation, differentiation, migration and immune responses.

Liver development is a complex process, encompassing the specialisation of the endoderm, the formation of hepatic progenitor cells, the emergence and proliferation of the liver bud, and ultimately, the determination of cell fate and maturation [85, 86]. Our research demonstrates that the composition and organisation of the liver ECM undergo substantial alterations during this process. Notably, in the neonatal liver, elevated expression levels of ECM proteins, such as Lumican, are closely associated with the maintenance of hepatic stemness and proliferative capacity. As aging progresses, the composition and structural organisation of the liver ECM evolve, changes that potentially lead to a decline in liver function. Age‐related alterations in the liver ECM could be linked to changes in the proportion of collagens, as well as an increase in ECM components associated with inflammation and fibrosis [87]. Our study reveals variations in the expression of ECM proteins in the livers of aged rats, potentially correlating with phenomena such as reduced liver volume, decreased blood flow, and compensatory hepatocyte hypertrophy.

The dynamic alterations in the liver ECM exert profound impacts on hepatocyte behaviour and functionality. Studies have demonstrated that various ECM components, such as fibronectin and laminin, significantly influence hepatocyte adhesion and proliferation [88, 89]. Our preliminary research, utilising matrisome and bioinformatics analyses, has revealed the pivotal roles of fibronectin and fibrinogen in enhancing hepatocyte maturation and functionality. Fibrinogen, in particular, facilitates cellular aggregation and strengthens cell–cell connections, thereby activating the Wnt/β‐catenin signalling pathway, essential for hepatocyte maturation [90]. Additionally, proteoglycans may play a pivotal bridging role within this complex network, interfacing with elastin and collagens in the matrix in a specific manner, thereby imparting unique structural integrity to the matrix [91, 92, 93]. Owing to the presence of heparan sulfate in most glycosaminoglycans, proteoglycans are widely distributed and play crucial regulatory roles in neural development, cell recognition, binding and differentiation [94, 95]. We have discovered that in the liver ECM, various proteoglycans such as Dcn, Lum, Prg2 and Prg3, play integral roles in regulating tissue development, signal transduction, metabolic processes, responses and reactions. Furthermore, we have demonstrated that Lumican can interact with multiple secretory protein factors, playing a vital role in neonatal liver development and regeneration.

Lumican plays a pivotal role in the pathogenesis and progression of various diseases. In ophthalmology, the absence of Lumican leads to conditions such as corneal opacification, glaucoma and myopia, highlighting its essential role in maintaining the normal structure of collagen fibres [96]. Lumican is also identified as a marker protein in pancreatic cancer and plays an integral role in the development and progression of both gastrointestinal and non‐gastrointestinal tumours, including liver, gastric, colorectal, lung, breast and prostate cancers [97, 98, 99]. It regulates cellular proliferation, migration and tumour growth. Furthermore, Lumican is involved in the recruitment and migration of neutrophils, interacts with Toll‐like receptor 1 to regulate immune responses, and mediates Fas–FasL activation to induce apoptosis, a critical process in inflammatory diseases such as Behçet's syndrome and infections with Pseudomonas aeruginosa [100]. In epithelial repair, Lumican modulates the cell cycle and promotes cell migration and wound healing through the TGF‐β signalling pathway [101, 102]. In our research, we have demonstrated that Lumican, by interacting with the hepatic cell surface receptor ITGA2, activates the ERK and mTOR signalling pathways, thereby promoting hepatocyte proliferation and maintaining stem cell characteristics, offering novel strategies for liver regeneration.

At the forefront of contemporary liver research, the role of ECM is increasingly acknowledged for its profound impact on liver health and disease states [90, 103, 104]. With advancements in single‐cell sequencing technologies and the integrative application of systems biology approaches, research into the ECM has evolved from focusing solely on intracellular transcriptional regulation to encompassing global regulatory mechanisms of the extracellular microenvironment [105, 106]. The establishment and advancement of matrisomics have provided powerful tools for decoding the complexities of the ECM [107, 108]. The findings of this study are closely aligned with the latest advancements in the field, emphasising the multidimensional role of the ECM in regulating liver development, regeneration and aging. By systematically analysing the variations in the ECM of rat livers across different age groups, we have elucidated the pivotal role of the ECM in liver aging and established a solid foundation for future research and clinical practice in this area. Our work offers a novel perspective on the functions of the ECM in liver development and aging, identifies potential therapeutic targets, and paves the way for the development of innovative intervention strategies.

In summary, this study, utilising quantitative proteomics, has elucidated the pivotal role of the ECM in liver aging and underscored the importance of a comprehensive understanding of ECM composition for the advancement of bioengineering and regenerative medicine strategies. Although our understanding of the liver ECM has progressed, further research is essential to elucidate the specific mechanisms through which the ECM influences liver aging. Future studies should concentrate more on the composition and functionality of the ECM, as well as its interactions with cell surface receptors, to comprehensively elucidate the role of the ECM in liver health and disease.

## 4 Materials and Methods

### 4.1 Animal Models and Preparation of Hepatic Biomatrix Scaffolds

Male Sprague–Dawley rats were obtained from Vital River Company (Beijing, China) and maintained under controlled pathogen‐free conditions at the Laboratory Animal Center of Beijing Tsinghua Changgung Hospital. The care and experimental procedures involving these animals adhered strictly to established guidelines for the ethical use and treatment of laboratory animals. For the preparation of liver‐specific biomatrices, rats were anaesthetised using a ketamine‐xylazine mixture. The livers were then decellularised using a perfusion technique previously described in our research [109, 110]. Subsequently, the decellularised liver scaffolds were pulverised into fine powder using a Freezer/Mill (SPEX Sample Preparation) in a liquid nitrogen environment. Protein concentrations within these samples were measured using the super Bradford protein assay kit (CWBIO). Following analysis, samples were preserved at ‐80°C for subsequent experimental analysis.

### 4.2 Characterisation of Liver Biomatrix Scaffolds

The efficacy of the decellularisation process was evaluated by assessing the retention of nucleic acid residues, collagens, and sulfated glycosaminoglycans (GAGs). To confirm the absence of cellular or cell debris residues, DNA content was quantified using a Fluorescent DNA Quantitation Kit (Bio‐Rad). Collagens and sulfated GAGs levels were assessed to indicate changes in the structural composition of the liver biomatrix scaffolds from rats of varying ages. A Silica dye collagen assay (Biocolour) was utilised to determine collagen levels by measuring absorbance at 555 nm. Similarly, sulfated GAGs were quantified using a Blyscan dye (Sulfated glycosaminoglycan assay, Biocolour) with absorbance readings at 656 nm, and results were normalised against a sodium heparin standard curve. All procedures were conducted strictly in accordance with the protocols recommended by the manufacturers.

Additionally, liver biomatrix scaffolds and normal liver tissues were fixed for histological examination. Paraffin‐embedded sections were processed according to a standard haematoxylin and eosin (H&E) staining protocol to evaluate the removal of cellular components. To assess the retention of matrix components within the scaffolds, immunohistochemical (IHC) staining was performed on the sections using specific antibodies.

### 4.3 Protein Extraction, Proteomic Analysis With LC–MS/MS and Bioinformatics Analysis

Protein extraction from liver biomatrix scaffolds was performed using a Type 4 Protein Extraction Reagent kit (C0356‐1BTL, Sigma). Following extraction, the proteins underwent reduction, alkylation and digestion with trypsin. The resultant peptides were subsequently labelled according to the specifications of the iTRAQ Reagent‐8 plex Multiplex Kit (AB SCIEX). These labelled peptides were analysed via LC–MS/MS on a Q Exactive HF Mass Spectrometer (Thermo Fisher Scientific), equipped with a nanospray source and operated alongside Eksigent high‐performance liquid chromatography.

The generated raw data files were processed using MaxQuant software (version 1.5.8.3). The MS/MS spectra were searched against the complete rat protein dataset from the UniProt database, using trypsin with allowance for up to two missed cleavages. Specific modifications included fixed cysteine carbamidomethylation (C+ 57.022), variable methionine oxidation (M+ 15.99491), and acetylation at the protein N‐terminus. A false discovery rate of 1% was maintained at both peptide and protein levels to ensure accuracy.

For bioinformatics analysis, ECM proteins were classified into core ECM (such as collagens, proteoglycans and ECM glycoproteins) and associated proteins (including ECM regulators, affiliated proteins and secreted factors) using the Matrisome DB. Further annotation of identified proteins based on Gene Ontology biological processes and KEGG/reactome signalling pathways was conducted using the OmicShare tools online. Additionally, a protein–protein interaction network was constructed using Cytoscape software (version 3.10.1), with interaction data retrieved from the STRING database, facilitating a comprehensive understanding of the proteomic landscape and its implications in liver biomatrix scaffolds.

### 4.4 Isolation and Culture of Primary Hepatocytes From Rat Neonatal Liver

Primary hepatocytes were isolated from the neonatal liver of C57BL/6J female mice on gestational days 12–14. The mice were euthanised via cervical dislocation, then sterilised with 70% ethanol and subjected to abdominal incision to extract the neonatal liver. Hepatocytes were dissociated by digestion in a 0.01% Type V collagenase solution at 37°C for 6 min, followed by centrifugation at 1500 rpm for 5 min. Cells were further dissociated using accutase enzyme until a single‐cell suspension was achieved, which was then collected by centrifugation. The cells were resuspended in PBS containing 2% foetal bovine serum, filtered through a 70 μm mesh, and subsequently centrifuged to purify the cell population. The supernatant was discarded, and cells were lysed using Buffer RLT; the lysate was subsequently transferred to a 1.5 mL centrifuge tube and stored at −80°C for subsequent RNA extraction.

For cell culture, 10 cm culture dishes and 24‐well plates were pre‐coated with 0.1% gelatin and incubated at 37°C for 10 min, after which the excess liquid was aspirated. Cells were resuspended in Kubota Medium (KM) supplemented with 8% foetal bovine serum and seeded at a density of 2 × 10^5^ cells per square centimetre in culture dishes and 24‐well plates. The cells were cultured at 37°C in a 5% CO_2_ atmosphere. After three days, the cells underwent immune‐fluorescence staining analysis. The preparation of KM involved dissolving RPMI 1640 powder in deionised water, adding albumin, nicotinamide, zinc sulfate, hydrocortisone, transferrin, insulin and free fatty acids, and adjusting the volume to 1 L.

### 4.5 Culture of HepaRG Cell Line

The human hepatoma cell line, HepaRG, was cultured in William's E medium (Gibco, USA), which was enriched with 10% foetal bovine serum (FBS, Gibco, Canada), penicillin (100 IU/mL), streptomycin (100 μg/mL), GlutaMax (2 mM, Gibco, USA), insulin (5 μg/mL) and hydrocortisone hemisuccinate (0.5 mM). The cells underwent subculturing at intervals of every two to three days.

### 4.6 Cell Proliferation Assays

To assess the impact of Lumican on hepatocyte proliferation, HepaRG cells were resuspended and enumerated in William's E medium supplemented with 10% FBS following trypsinisation, then seeded in 24‐well and 96‐well plates and cultured at 37°C in a 5% CO_2_ atmosphere. The following day, cells were serum‐starved for 24 h and subsequently treated with various concentrations of Lumican. Cell proliferation rates over 24 h were quantified using the CCK8 assay, and DNA synthesis capacity was evaluated through EdU incorporation experiments. Following fixation, bleaching and permeabilisation, cells were stained with Apollo reaction solution for EdU labelling, and subsequently stained with Hoechst 33342 for fluorescence microscopy analysis to determine the positivity rate.

For the cellular pathway study, following serum starvation, HepaRG cells were treated with a mixture of small molecule inhibitors (20 nM PF431396, 10 μM LY294002, 10 μM U0126, 10 nM Torin1, 0.1 μg/mL E7820) and 30 ng/mL Lumican in the culture medium. Cell proliferation was assessed using both the CCK8 and EdU staining methods.

### 4.7 Immunohistochemistry (IHC) and Immunofluorescence (IF) Staining Procedures

Liver tissues and scaffolds were preserved in 4% paraformaldehyde (PFA), embedded in paraffin and sectioned into 5 μm slices. For IHC, the paraffin‐embedded sections underwent rehydration, were treated with an antigen retrieval solution, and blocked with serum. The staining process was conducted using a Vector kit (Vector Laboratories) as per the instructions provided by the manufacturer. The specific antibodies applied are detailed in Table S1.

For IF staining, cells fixed in 4% PFA were permeabilised using 0.2% Triton X‐100 and blocked with 10% serum from either goat or donkey. These were subsequently incubated with primary antibodies at 4°C overnight. Secondary antibodies were applied for one hour in a dark environment at room temperature, followed by a 4′6‐diamidino‐2‐phenylindole (DAPI) staining for visualisation of nuclei. Imaging of cultured cells, spheroids and organoids was performed using an Operetta High Content Imaging System (PerkinElmer).

### 4.8 Quantitative Real‐Time PCR (qRT‐PCR) Analysis

Total RNA from spheroids was extracted using a RNeasy mini kit (Qiagen). This RNA was then converted to cDNA employing the ReverTra Ace qPCR RT Master Mix (Toyobo), following the protocol recommended by the manufacturer. The qRT‐PCR assays were conducted using a Bio‐Rad iQ5 Real‐Time PCR Detection System (Bio‐Rad) and SYBR green master mix (Toyobo). Expression levels of target genes were quantified relative to the housekeeping gene GAPDH using the 2^(‐ΔΔCt) method. Details of the primers utilised are provided in Supplementary Table S2.

### 4.9 Western Blotting and co‐Immunoprecipitation (Co‐IP)

Cells were lysed using a buffer (Beyotime Biotechnology) supplemented with protease and phosphatase inhibitors (Roche). The lysates were subjected to sonication for 30 s, followed by centrifugation at 12,000 rpm for 10 min at 4°C. Proteins were resolved through 6%–10% sodium dodecyl sulfate‐polyacrylamide gel electrophoresis and subsequently transferred onto polyvinylidene difluoride membranes. The membranes were then incubated with primary antibodies and horseradish‐peroxidase (HRP)‐conjugated anti‐rabbit IgG antibodies (refer to Table S1 for details). Detection of target proteins was performed using an enhanced chemiluminescence HRP substrate (Millipore).

When HepaRG cells grew to 80%–90% confluent in a 10 cm dish, the cells were digested by 0.25% pancreatic enzyme, centrifuged at 1000 rpm for 3 min, collected into a 2 mL centrifuge tube, and pre‐cooled protein lysate was added (1 mL protein lysate was added every 3 dishes). The cells were broken on the ice with an ultrasonic cell crusher, centrifuged at 4°C 10000 g for 10 min, and the supernatant was transferred to a clean 1.5 mL centrifuge tube. The cell protein samples were evenly divided into two parts; one was the control part, adding 1 μg control immunoglobulin, and the other was added 1‐2 μg monoclonal protein. Both were mixed with magnetic beads and incubated at 4°C on the rocker arm rotator overnight. The samples incubated overnight were centrifuged at 4°C 1000 g for 5 min and the supernatant was discarded. Add 1 mL of protein lysate and rotate at 4°C on the rocker arm rotator for 10 min to clean the magnetic beads. Repeat cleaning 2–3 times, re‐suspending magnetic beads with 2 × Loading Buffer, cook for 10 min at 100°C, and store the packaged protein at −20°C.

For the concentration gradient experiment, HepaRG cells were treated with 0–300 ng/mL Lumican for 12 h. For the time gradient experiment and Co‐IP, the Lumican concentration was fixed at 30 ng/mL, and they were treated for the corresponding times respectively.

### 4.10 Protein–Protein Interaction Network Analysis

Map the ligand receptor molecules of ECM and ECM to the STRING database (https://string‐db.org/) The protein–protein interaction network (PPI) was established with a medium confidence value of 0.400. Then import Cytoscape software, use ForceAtlas2 layout optimisation visualisation, adjust the node colour (corresponding to the legend classification). In the PPI network, the nodes represent the target protein, and the lines represent the protein interaction defined by STRING, whose thickness is positively correlated with the interaction confidence.

### 4.11 Statistical Analysis

Unless specified otherwise, all experiments were conducted in triplicate. Standard deviations are denoted by error bars. The data were expressed as mean ± standard deviation (SD), based on three independent experiments. The Student's t‐test was employed for the statistical evaluations. Data visualisation was performed using Origin 9.0 and GraphPad Prism 8 software.

## Author Contributions

J.L. and Y.W. offered main direction and significant guidance of this manuscript. J.L. and Q.S. drafted the manuscript. Q.S. and C.L. conducted proteomics experiments, data collection and preliminary analysis. J.Y., N.A. and W.Y. were involved in the preparation of liver decellularised scaffolds and immunohistochemical experiments. J.D. and Y.S. are responsible for bioinformatics analysis and result interpretation. All authors approved the final manuscript.

## Conflicts of Interest

The authors declare no conflicts of interest.

## Supporting information


**Data S1.** Supporting Information.

## Data Availability

All data associated with this study are present in the paper or in the Supporting Information.
