# Supplementary material for: Deciphering Age‐Dependent ECM Remodelling in Liver: Proteomic Profiling and Its Implications for Aging and Therapeutic Targets
Source: Cell Prolif. 2025 Jun 19;58(9):e70087. doi: 10.1111/cpr.70087 (PMC12414644; doi:10.1111/cpr.70087)
Supplement: Supplementary file 1 — Data S1. Supporting Information. [file CPR-58-e70087-s001.docx]

**Supporting information**

**Deciphering Age-Dependent ECM Remodeling in Liver: Proteomic Profiling and Its Implications for Aging and Therapeutic Targets**


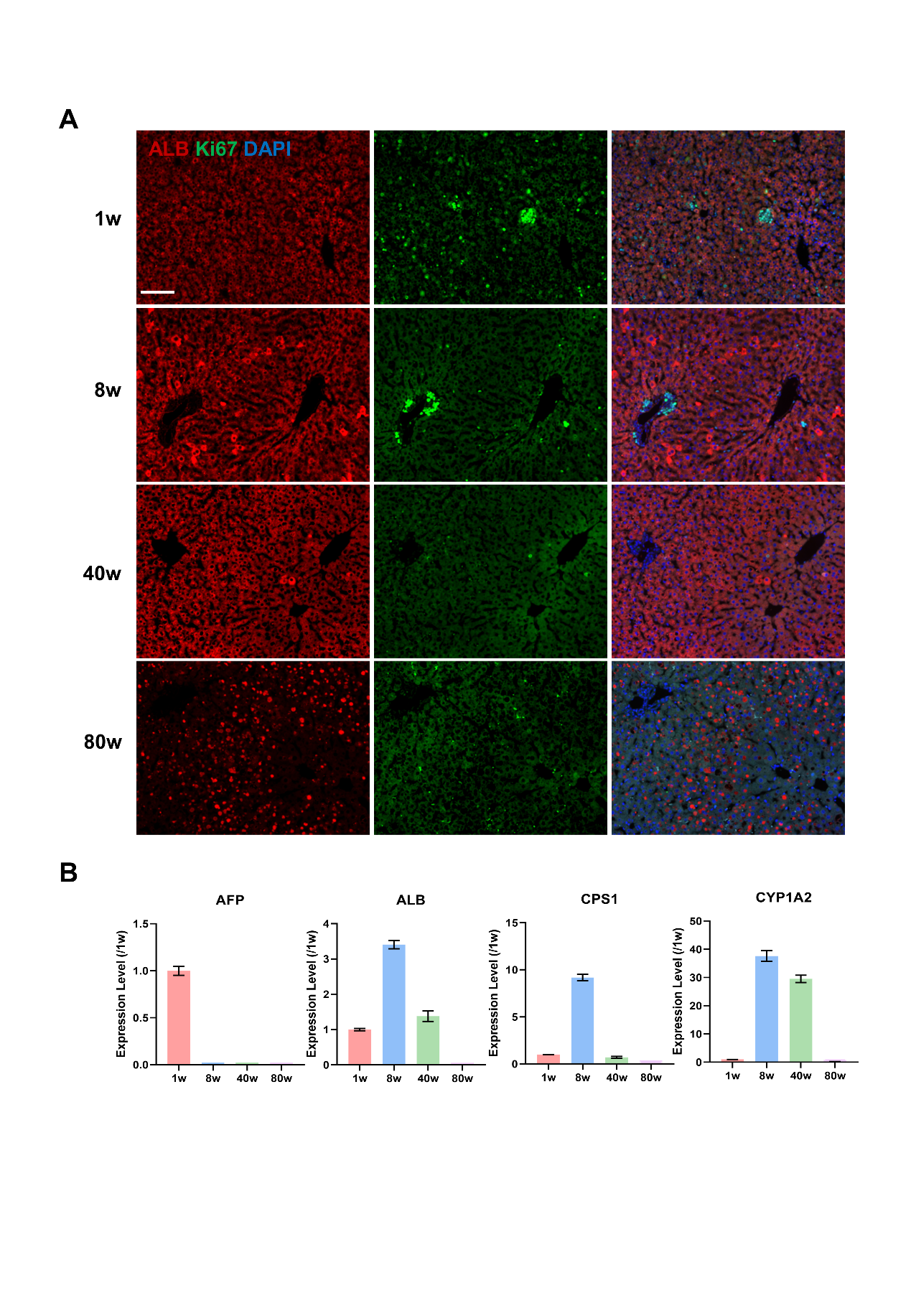


**Figure S1. (A) Fluorescence expression of Ki67 and ALB in liver tissue of rats at different age stages.** Scale bar =100μm. (B) **Expression of liver function-related indicators and drug metabolism gene in liver tissue of rats at different age stages.**


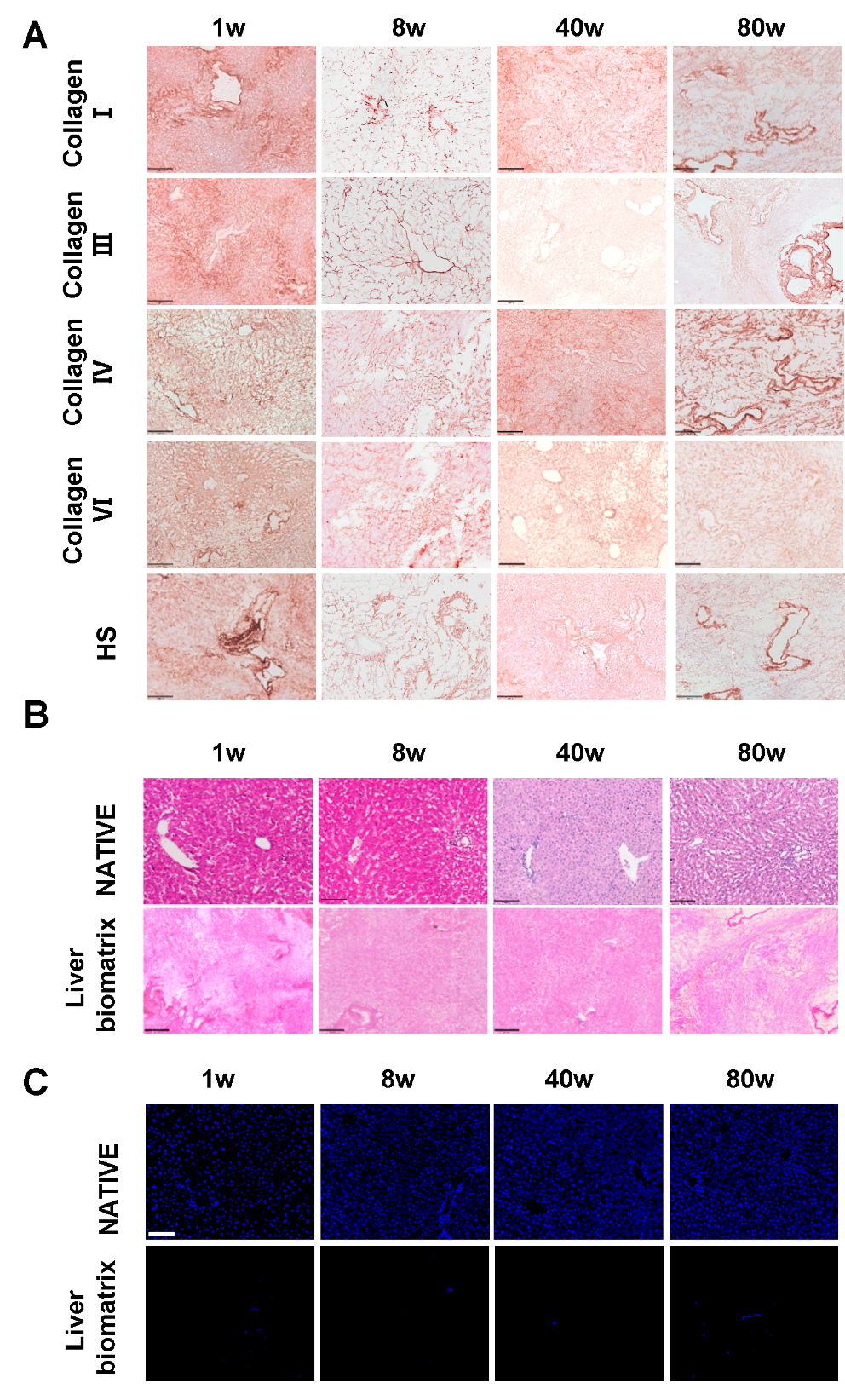


**Figure S2**. (A) IHC staining results of major protein components in decellularized liver bio-scaffolds from rats at different age stages. (B) HE staining results of native liver and decellularized liver bio-scaffolds from rats at different age stages. (C) DAPI staining results of native liver and decellularized liver bio-scaffolds from rats at different age stages, which are used to detect the presence of nuclear material and confirm the absence of cellular components. Scale bar =100μm.


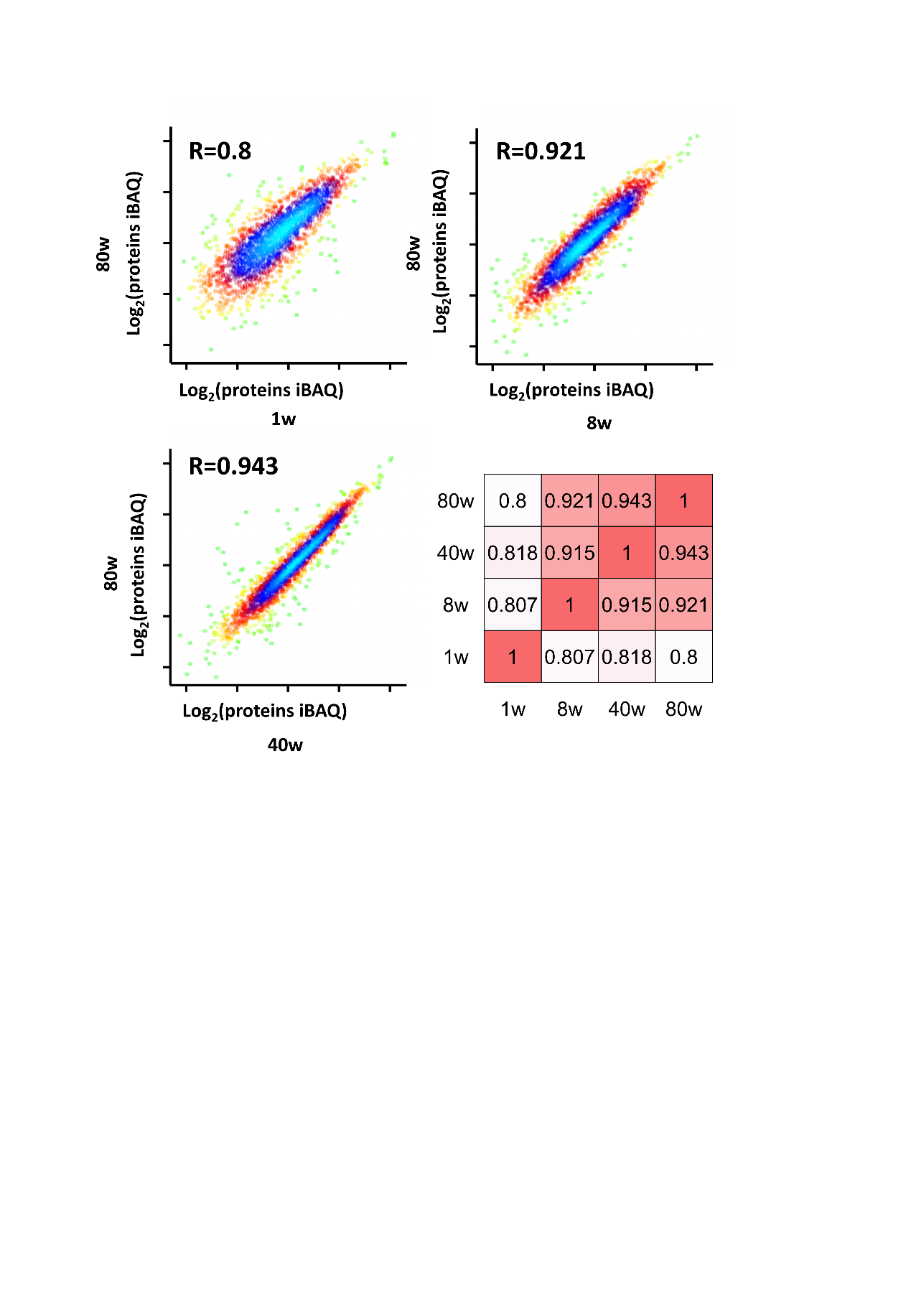


**Figure S3.** Correlation analysis of proteomic profiles in decellularized liver scaffolds across age groups. The plots show the Log2-transformed protein quantities in iBAQ units, with correlation coefficients (R values) on each, indicating the relationship strength between age groups.


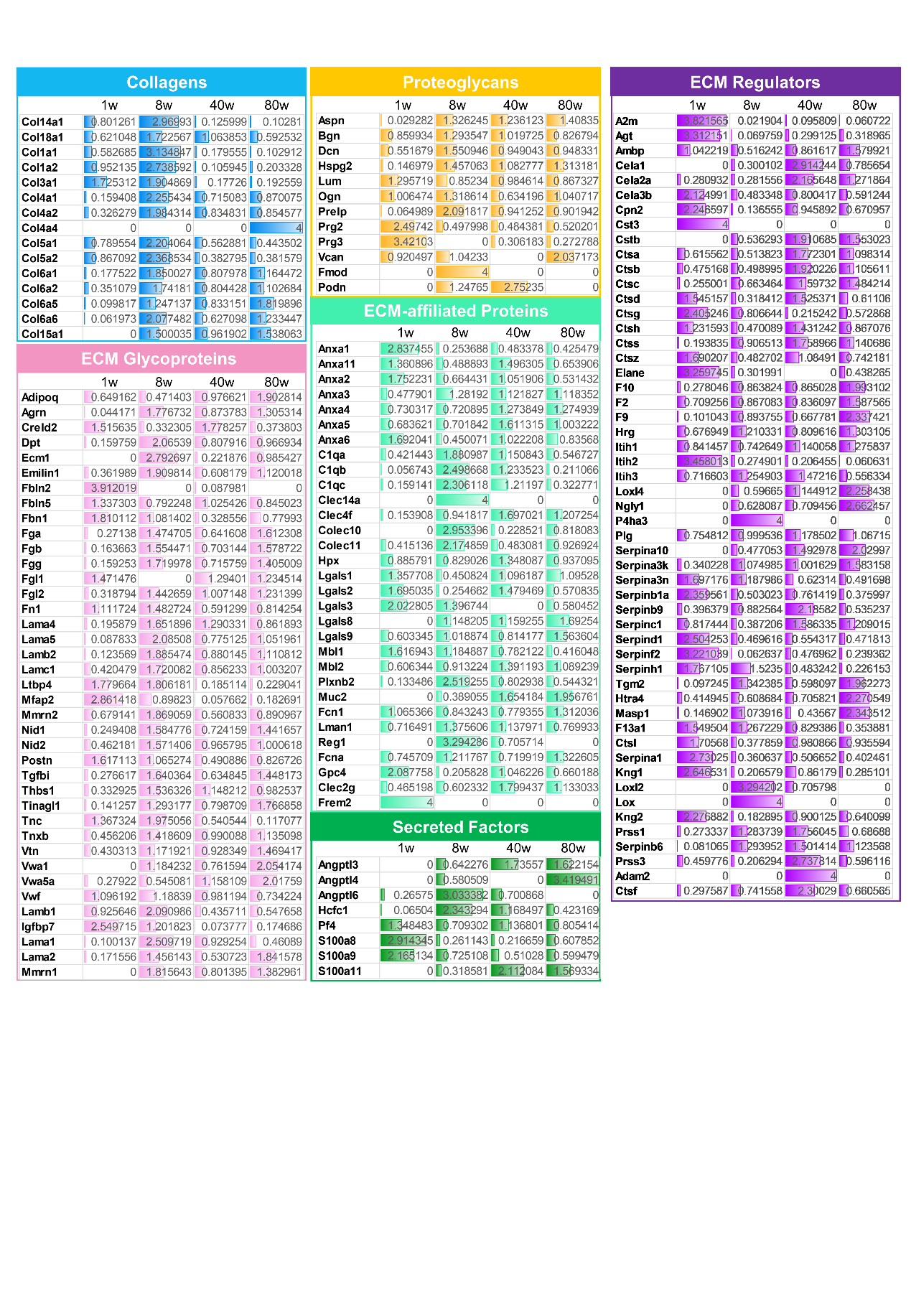


**Figure S4.** Relative expression levels of ECM molecules in rat liver across different age stages.


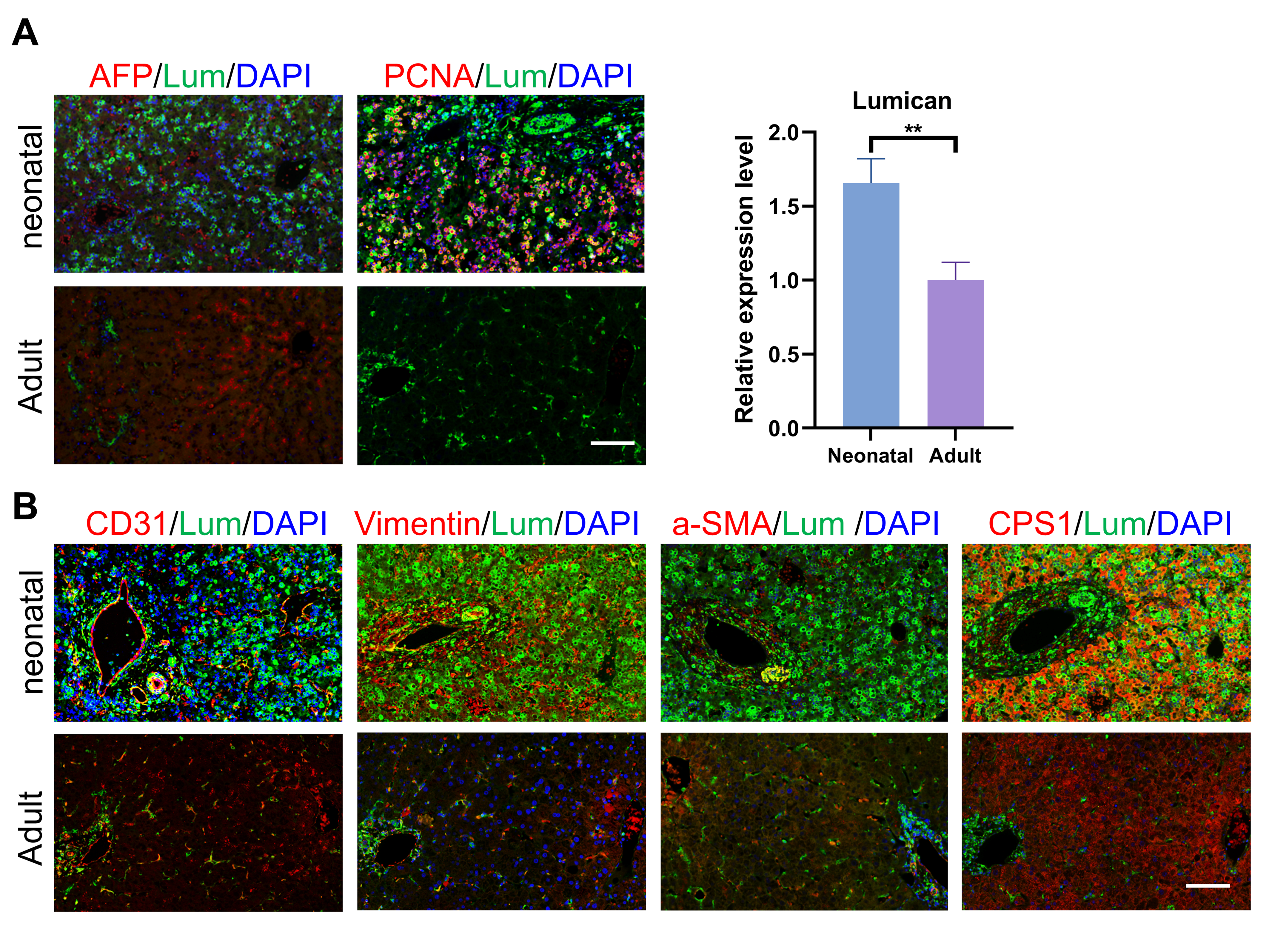


**Figure S5.** Lumican was highly expressed in juvenile liver tissue. (A) Q-PCR analysis of Lumican in juvenile and adult rat hepatocytes. (B) Immunofluorescence expression of Lumican in various hepatocyte subtypes of juvenile liver. Scale bar =100μm. **, p≤0.01; two-tailed Student’s t-tests. All experiments were run in triplicate.


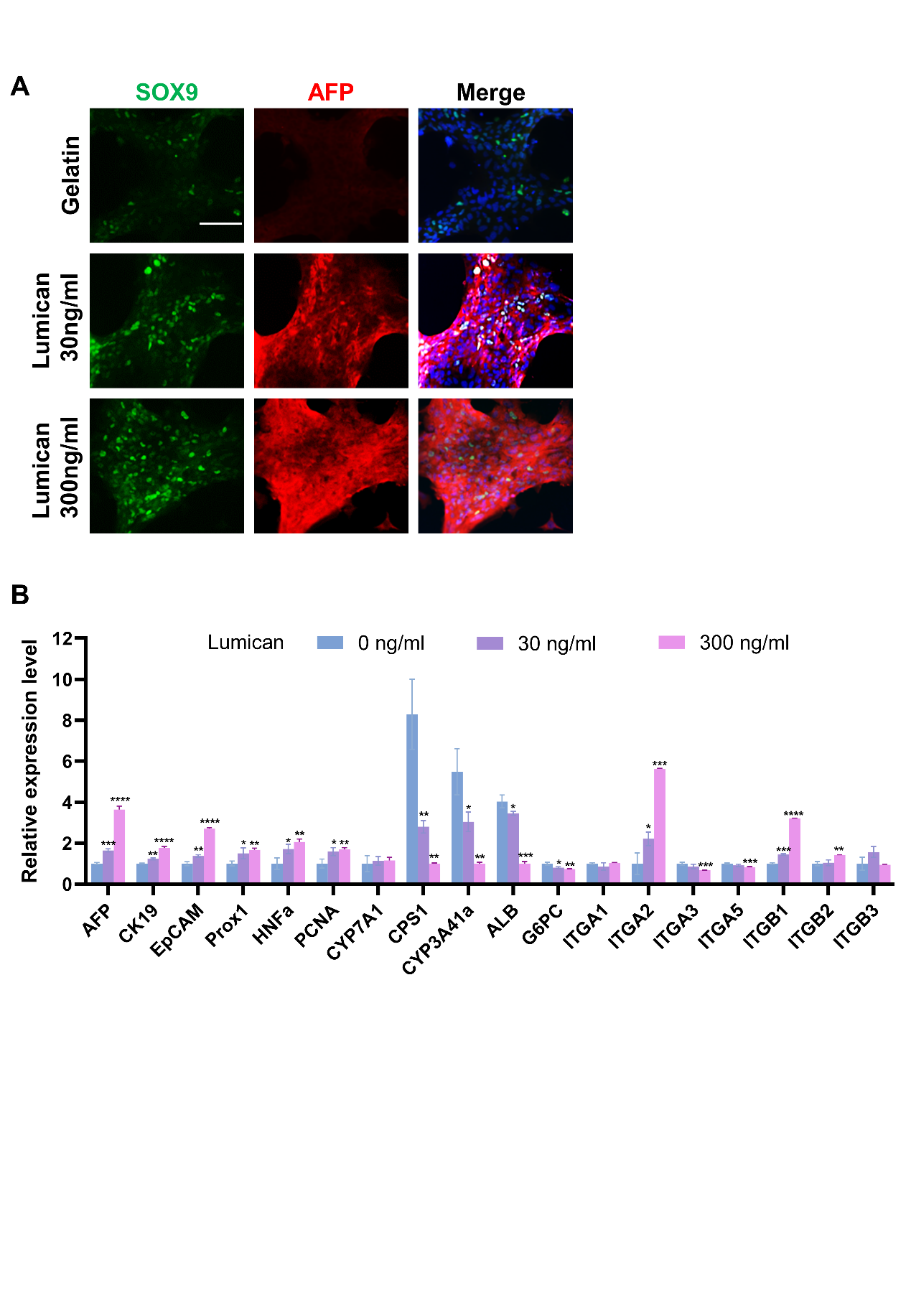


**Figure S6.** Lumican plays an important role in maintaining neonatal hepatocyte stemness. (A) Increased Lumican concentrations at different treatments, immunofluorescence results of liver stem cell markers SOX9 and AFP. Scale bar =100μm. (B) Q-PCR results of liver mature cell markers and liver stem cell markers in neonatal hepatocytes treated with different Lumican concentrations for 12h. *, p≤0.05; **, p≤0.01; ***, p≤0.001; ****, p≤ 0.0001; two-tailed Student’s t-tests. All experiments were run in triplicate.


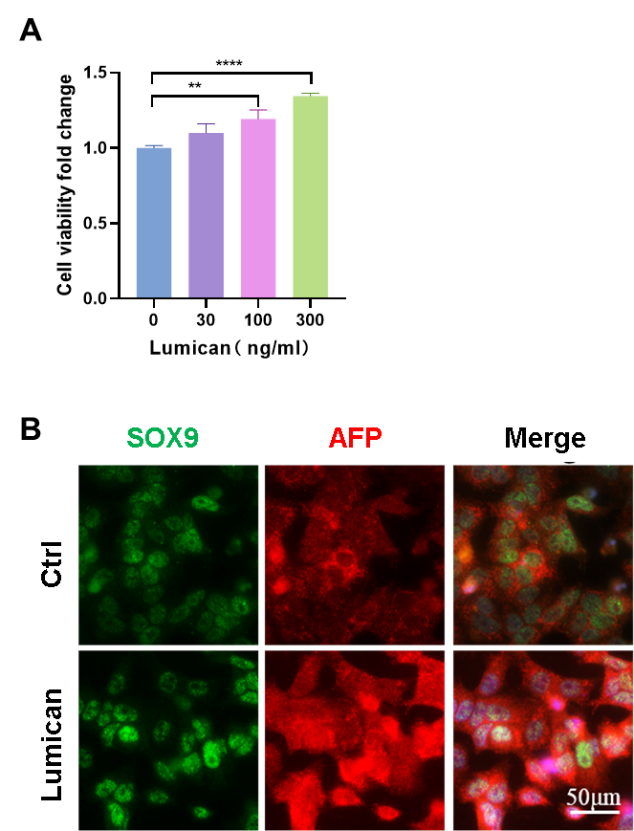


**Figure S7.** The effects of Lumican on the proliferation ability and stemness maintenance of HepaRG cells. (A) Changes in proliferation capacity of HepaRG cells under different concentrations of Lumican. (B) Immunofluorescence results of liver stem cell marker SOX9 and AFP before and after Lumican treatment (30ng/ml). Scale bar =50μm. **, p≤0.01; ****, p≤ 0.0001; two-tailed Student’s t-tests. All experiments were run in triplicate.


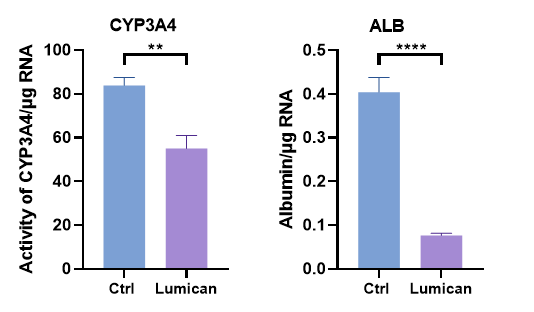


**Figure S8.** The effects of Lumican (30ng/ml) on HepaRG cell sphere liver function. **, p≤0.01; ****, p≤ 0.0001; two-tailed Student’s t-tests. All experiments were run in triplicate.


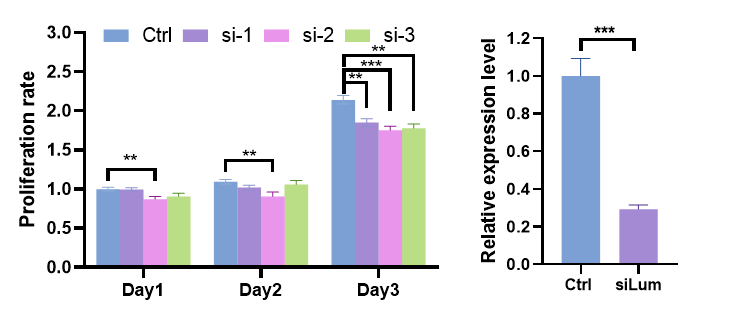


**Figure S9.** The effect of the absence of Lumican on the proliferation of HepaRG cells. ***, p≤0.001; two-tailed Student’s t-tests. All experiments were run in triplicate.

**Table S1.** Information for Antibodies Used in Western Blots (WB), Immunohistochemistry (IHC) and immunofluorescence (IF).

| Name | Vendor | Catalog No. | Species | Dilution |
| --- | --- | --- | --- | --- |
| Fibronectin | abcam | ab268020 | rabbit | 1:200 |
| Annexin Ⅳ | MedChemExpress | YA2714 | rabbit | 1:100 |
| Lumican | abcam | ab168343 | rabbit | 1:200 |
| CK19 | abcam | ab52625 | rabbit | 1:200 |
| ALB | abcam | ab19194 | goat | 1:200 |
| Ki67 | Thermo | 13-5698-82 | rat IgG2a | 1:100 |
| P70S6K | Santa Cruz | sc-8418 | rat IgG1 | 1:200 |
| CCND1 | Santa Cruz | sc-8396 | rat IgG2b | 1:200 |
| GAPDH | ProteintechTM | HRP-6004 | rat IgG2b | 1:200 |
| pERK | Santa Cruz | sc-136521 | rat IgG1 | 1:200 |
| ITGA2 | Millipore | MAB1950Z | rat IgG1 | 1:200 |
| CollagenⅠ | abcam | ab270993 | rabbit | 1:100 |
| Collagen Ⅲ | Proteintech | 22734-1-AP | rabbit | 1:200 |
| Collagen Ⅳ | abcam | ab236640 | rabbit | 1:100 |
| Collagen Ⅵ | Proteintech | 17023-1-AP | rabbit | 1:200 |
| AFP | SIGMA | A8452 | rat IgG2a | 1:200 |
| PCNA | CST | 2586 | rat IgG2a | 1:2500 |
| CD31 | abcam | ab9498 | rat IgG1 | 1:200 |
| Vimentin | abcam | ab8978 | rat IgG1 | 1:200 |
| a-SMA | abcam | ab7817 | rat IgG2a | 1:100 |
| CPS1 | Santa Cruz | sc-10515 | goat | 1:200 |
| SOX9 | abcam | ab185966 | rabbit | 1:200 |

**Table S2.** Information for Primer sequence Used in Quantitative Polymerase Chain Reaction (Q-PCR).

| Gene | Forward primer | Reverse primer |
| --- | --- | --- |
| AFP | CTTCCCTCATCCTCCTGCTAC | ACAAACTGGGTAAAGGTGATGG |
| ALB | TGCTTTTTCCAGGGGTGTGTT | TTACTTCCTGCACTAATTTGGCA |
| CCND1 | TCTGGCTATTTTAGTTGCCACAG | GCCTGACCAAATTCGTACCTG |
| CDC20 | GCACAGTTCGCGTTCGAGA | CTGGATTTGCCAGGAGTTCGG |
| CDC25A | GTGAAGGCGCTATTTGGCG | TGGTTGCTCATAATCACTGCC |
| CK19 | GGGGGTTCAGTACGCATTGG | GAGGACGAGGTCACGAAGC |
| C-MYC | TACAACACCCGAGCAAGGAC | TTCTCCTCCTCGTCGCAGTA |
| CPS1 | ACATGGTGACCAAGATTCCTCG | TTCCTCAAAGGTGCGACCAAT |
| CYP3A41a | TGGACAGAATGAAGGAAAGCC | AATTGACTGGGCTGTGATCTC |
| CYP7A1 | GGGATTGCTGTGGTAGTGAGC | GGTATGGAATCAACCCGTTGTC |
| ETV5 | AGGGGCAGAAAACCACCAAA | GTCCCGTTTTGCGGGTACTA |
| EрCAM | GCGGCTCAGAGAGACTGTG | CCAAGCATTTAGACGCCAGTTT |
| FOXM1 | ATAGCAAGCGAGTCCGCATT | TTCCTCCCCAGGCTGGATTT |
| G6PC | CGACTCGCTATCTCCAAGTGA | GTTGAACCAGTCTCCGACCA |
| GAPDH | AGGTCGGTGTGAACGGATTTG | TGTAGACCATGTAGTTGAGGTCA |
| HNF4a | GCCACCACTGGTGACCTT | CCTTCTGCTGTTGCTGCTG |
| ITGα1 | CCAAACATGTCTTCCACCG | CTGCTGCTGGCTCCTCAC |
| ITGα2 | ACTCAAGCTACCACACACCG | TGTGTATCGATCTCTGCCGC |
| ITGα3 | GGTTGGTGTAGCCATCGG | CCTCTTCGGCTACTCGGTC |
| ITGα5 | AGGTAGACAGCACCACCCTG | CTCAGTGGAGTTTTACCGGC |
| ITGβ1 | GGACGCTGCGAAAAGATGAA | CCACAATTTGGCCCTGCTTG |
| ITGβ2 | TCCCAGGAATGCACCAAGTACAA | CAGTGAAGTTCAGCTTCTGGCA |
| ITGβ3 | GTGACCTGAAGGAGAATCTGC | CCGGAGTGCAATCCTCTGG |
| Lumican | CTCTTGCCTTGGCATTAGTCG | GGTCATCACAGTACATGGCAGT |
| PCNA | TTTGAGGCACGCCTGATCC | GGAGACGTGAGACGAGTCCAT |
| Prox1 | AGAAGGGTTGACATTGGAGTGA | TGCGTGTTGCACCACAGAATA |
| TGF-β | GGCCAGATCCTGTCCAAGC | GTGGGTTTCCACCATTAGCAC |
